# Enantioselective synthesis, characterization, molecular docking simulation and ADMET profiling of α-alkylated carbonyl compounds as antimicrobial agents

**DOI:** 10.1038/s41598-024-61884-9

**Published:** 2024-05-21

**Authors:** Ahmed A. Noser, Mariam Ezzat, Shimaa G. Mahmoud, Adel I. Selim, Maha M. Salem

**Affiliations:** 1 Chemistry Department, Faculty of Science, Tanta University, Tanta, 31527 Egypt; 2 Biochemistry Division, Chemistry Department, Faculty of Science, Tanta University, Tanta, 31527 Egypt

**Keywords:** Asymmetric synthesis, Enantiomeric pure, HPLC, OMPA, Exo-1,3-beta-glucanase, Chemical biology, Chemistry

## Abstract

All living organisms produce only one enantiomer, so we found that all natural compounds are presented in enantiomerically pure form. Asymmetric synthesis is highly spread in medicinal chemistry because enantiomerically pure drugs are highly applicable. This study initially demonstrated the feasibility of a good idea for the asymmetric synthesis of α-alkylated carbonyl compounds with high enantiomeric purity ranging from 91 to 94% using different quinazolinone derivatives. The structure of all compounds was confirmed via elemental analysis and different spectroscopic data and the enantioselectivity was determined via HPLC using silica gel column. The synthesized compounds’ mode of action was investigated using molecular docking against the outer membrane protein A (OMPA) and exo-1,3-beta-glucanase, with interpreting their pharmacokinetics aspects. The results of the antimicrobial effectiveness of these compounds revealed that compound **6a** has a broad biocidal activity and this in-vitro study was in line with the in-silico results. Overall, the formulated compound **6a** can be employed as antimicrobial agent without any toxicity with high bioavailability in medical applications.

## Introduction

According to the World Health Organization (WHO), antimicrobial resistance (AMR) is expected to be a factor in 10 million deaths per year by 2030, with an additional consequence of 24 million people living in absolute poverty. Treating pathogenic infections caused by multi-drug-resistant microorganisms is a significant challenge, particularly for individuals with impaired immune systems. The primary possibilities for achieving this goal are the discovery of novel antimicrobial bioactive chemicals and/or the reconfiguration of currently available antimicrobial medications^1,2^.

Quinazolinones are a significant class of heterocyclic compounds with a wide range of biological activity. The quinazolinone derivatives are also an important class of chemicals for synthetic organic chemistry due to their straightforward production and potential for usage in asymmetric applications^3–5^.

One of the most significant reactions in organic synthesis and one that has greatly influenced the growth of organic chemistry is the formation of a new carbon-carbon bond alpha (α) to a carbonyl group. Ketones and carboxylic acids offer a wide range of applications in this regard. This is undoubtedly a result of the variety of enolate chemistry formed from ketones and acids and the quantity of α- substituted carbonyl compounds in physiologically active systems, but it is also a result of the wide range of starting materials. Asymmetric substitution of carbonyls has received a lot of attention since it frequently results in a new stereogenic center^6–9^. A class of chemicals known as α-alkylated ketones and acids includes substances with a wide range of biological functions and are also important intermediates in the production of other chemicals. The conventional method for making these compounds is the α-alkylation of carbonyl compounds using alkyl halides as the alkylating agents in the presence of an inorganic strong base^10,11^.

**Figure 1:**
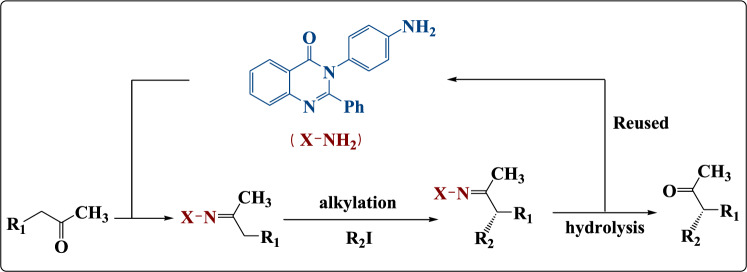
Asymmetric synthesis of α-alkylated ketone.

**Figure 2:**
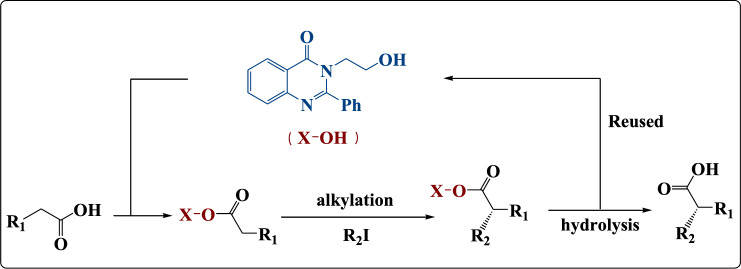
Asymmetric synthesis of α-alkylated carboxylic acid.

In the recent work, we described the asymmetric synthesis of α-alkylated ketone using 3-(4-aminophenyl)-2-phenylquinazolin-4(3H)-one (Fig. 1). In addition, we described the asymmetric synthesis of α-alkylated carboxylic acid using 3-(2-hydroxyethyl)-2-phenylquinazolin-4(3H)-one as described in Fig. 2. Also, these quinazolinone derivatives were evaluated in-silico against the bacterial outer membrane protein A (OMPA) and the fungus exo-1,3-beta-glucanase. After that, to widen their use as antimicrobial agents, the in-silico results were confirmed by examining their inhibitory activity in-vitro towards three multi-drug-resistant strains of bacteria and two pathogenic fungus strains.

## Experimental section

### Materials and instrumentation

All reagents of analytical quality were purchased from Sigma-Aldrich. All melting points were measured without adjustments on Gallen Kamp melting point equipment. On a Perkin-Elmer FTIR 1430 spectrophotometer, the Fourier transform infrared spectroscopy (FTIR) spectra were captured. On a JEOL AC spectrometer (400 MHz), the ^1^H nuclear magnetic resonance (NMR) spectra were captured at 25 °C in DMSO-d_6_, with TMS serving as an internal standard. Chemical shifts were given in parts per million as values, and the ^13^C NMR was set at 101 MHz. Elemental investigations for C, H, and N were also carried out, and the outcomes were found to be within 0.4% of theoretical values. Thin layer chromatography (TLC) was employed to monitor the reaction’s development. The separation of diastereomers was carried out using the HPLC technique with a silica gel column, eluent, petroleum ether/ ethyl acetate 8:2, F (flow rate =1 mL/ min), detector: UV 254nm.

### Synthesis of 3-(4-aminophenyl)-2-phenylquinazolin-4(3H)-one (2)

Compound **2** was synthesized as described by Vijayakumar^12^.

### Synthesis of 3-(2-hydroxyethyl)-2-phenylquinazolin-4(3H)-one (3)

Compound **3** was synthesized as described by Yang^13^.

### General procedure for the synthesis of compounds 4a, b

A mixture of 3-(4-aminophenyl)-2-phenylquinazolin-4(3H)-one (**2**) (1.1 g, 3.5 mmol), ketones (3.8 mmol), anhydrous magnesium sulphate (0.5 g, 4.2 mmol), few drops of glacial acetic acid in ethanol (20 mL) was refluxed for 16 h (TLC control), the product precipitated by adding ice, filtrated off, dried to give **4a, b**.

#### (Z)-3-(4-(hexan-2-ylideneamino) phenyl)-2-phenylquinazolin-4(3H)-one (4a)

Brown powder; m.p 168 °C, yield 99%; IR (KBr) v/cm^−1^: 3090 (CH-arom), 2923 (CH-aliph), 1642 (C=O), 1534 (C=N); ^1^H NMR (DMSO-d_6_) δ ppm: 0.80 (t, 3H, CH_3_), 1.15-1.30 (m, 4H, 2CH_2_ ), 1.40 (t, 2H, CH_2_ ), 2.01 (s, 3H, CH_3_), 7.55–7.94 (m, 13H, Ar–H); ^13^C NMR (DMSO-d_6_) δ ppm: 16.30, 22.60, 26.00, 31.90, 39.82, 115.07, 117.15, 120.33, 123.38, 123.61, 127.55, 129.48, 131.82, 132.66, 134.73, 135.08, 141.70, 144.00, 152.00, 165.19, 170.65; elemental analysis calculated for C_26_ H_25_ N_3_ O (395.50); C, 78.96%, H, 6.37%, N, 10.62%; founded; C, 78.56%, H, 6.07%, N, 10.22%.

#### (Z)-3-(4-(pentan-2-ylideneamino) phenyl)-2-phenylquinazolin-4(3H)-one (4b)

Brown powder, m.p 195 °C, yield 92.5%; IR (KBr) v/cm^−1^ : 3096 (CH-arom), 2925 (CH-aliph), 1682 (C=O), 1587 (C=N); ^1^H NMR (DMSO-d6) ppm: 0.80 (t, 3H, CH_3_), 1.12 (m, 2H, CH_2_), 1.29 (t, 2H, CH_2_), 2.02 (s, 3H, CH_3_), 7.54–8.04 (m, 13H, Ar–H); ^13^C NMR (DMSO-d_6_) ppm: 12.00, 27.00, 29.00, 39.80, 114.60, 117.55, 120.30, 123.37, 123.70, 127.56, 129.49, 131.83, 132.65, 134.59, 135.12, 141.66, 146.20, 152.60, 165.19, 170.70; elemental analysis calculated for C_25_ H_23_ N_3_O (381.47); C, 78.71%, H, 6.08%, N, 11.02%; founded; C, 78.68%, H, 5.78%, N, 10.82%.

### General procedure for the synthesis of 3-(4-(3-ethylhexan-2-ylideneamino)phenyl)-2-phenylquinazolin-4(3H)-one (5a, b)

A mixture of compounds **4a** or **4b** (3.5 mmol) was dissolved in THF (20.0 mL), then cyclohexyl iso propyl amine (CHIPA) (0.6 mL, 6.0 mmol) was added at −96 °C, after that *n*-BuLi (0.4 mL, 6.0 mmol) was added drop wise with stirring, the reaction mixture was stirred for 1h and treated with alkyl halide (7.0 mmol), the reaction mixture was stirred over night at room temperature, the product was filtrated off and dried to give **5a, b**^4,14^**.**

Brown crystals; m.p 255 °C, yield 75–86%; IR (KBr) v/cm^−1^: 3024 (CH-arom), 2855 (CH-aliph), 1636 (C=O), 1596 (C=N); ^1^H NMR (DMSO-d_6_) δ ppm: 0.89 (t, 6H, 2CH_3_), 1.13-1.27 (m, 6H, 3CH_2_), 1.30 (m, 1H, CH), 1.98 (s, 3H, CH_3_), 7.54–8.54 (m, 13H, Ar–H); ^13^C NMR (DMSO-d_6_ ) δ ppm: 10.00, 12.00, 20.00, 24.00, 26.80, 30.00, 31.80, 114.14, 116.00, 122.06, 123.72, 127.46, 127.55, 129.51, 132.59, 134.00, 136.00, 140.00, 144.00, 145.80, 156.69, 164.90, 171.90; elemental analysis calculated for (C_28_H_29_N_3_O (423.55); C, 79.40%, H, 6.90%, N, 9.92%; Founded; C, 79.10%, H, 6.60%, N, 9.70%.

### Synthesis of 3-ethylhexan-2-one (6a, b)

A mixture of **5a, b** (2.1 g, 5.0 mmol) was dissolved in Dioxane (20.0 mL), then methane sulphonic acid (few drops) was added and refluxed for 12 h, the product precipitated by adding ice, filtrated off, then compounds **6a, b** were extracted from the filtrate via methylene chloride.

Yellow liquid; yield 86–88%; IR (KBr) v/cm^−1^: 2922 (CH-aliph), 1739 (C=O); ^1^H NMR (DMSO-d_6_) δ ppm: 0.81 (t, 6H, 2CH_3_)_,_ 1.19–1.41 (m, 6H, 3CH_2_), 1.95 (s, 3H, CH_3_), 2.19 (m, 1H, CH); ^13^C NMR (DMSO-d_6_ ) δ ppm: 10.00, 16.00, 17.90, 19.00, 29.00, 31.90, 55.41, 212.00 ; elemental analysis calculated for C_8_H_16_O (128.21); C, 74.94%, H, 12.58%; Founded; C, 74.50%, H, 12.30%.

### Synthesis of (2R)-2-(3-ethylhexan-2-ylideneamino)-2-phenylethanol (7a, b)

A mixture of compounds **6a, b** (2.0 g, 15.0 mmol), R-phenyl glycinol (2.05 g, 15.0 mmol), ethanol (20.0 mL) and few drops of glacial acetic acid was refluxed for 10 h. (TLC control), then the diastereomeric compounds were extracted with methylene chloride to give compounds **7a, b**.

Yellow Liquid, yield 84–86%; IR (KBr) v/cm^−1^: 3460 (OH), 3097 (CH-arom), 2920 (CH-aliph), 1600 (C=N). ^1^H NMR (DMSO-d_6_) δ ppm: 0.82 (t, 6H, 2CH_3_), 1.16-1.21 (m, 6H, 3CH_2_), 1.24 (m, 1H, CH), 1.83 (d, 2H, CH_2_), 2.01 (s, 3H, CH_3_), 3.43 (s, H, OH), 4.10 (t, 1H, CH), 7.52–7.33 (m, 5H, Ar–H). ^13^C NMR (DMSO-d_6_) δ ppm: 10.89, 13.86, 20.30, 21.10, 22.50, 29.50, 32.00, 60.44, 77.01, 126.78, 127.90, 128.80, 138.80, 171.47; elemental analysis calculated for C_16_H_25_NO (247.38); C, 77.68%, H, 10.19%, N, 5.66%; founded; C, 77.43%, H, 10.08%, N, 5.20%.

### General procedure for the synthesis of quinazolinone esters (8a, b)

In 100 mL round bottom flask a mixture of compound **3** (2.6 g, 10.0 mmol), carboxylic acid derivatives (10.0 mmol), methane sulphonic acid (few drops) and 50.0 mL benzene was refluxed for 4 h using Dean–Stark trap (TLC control), then the reaction mixture was basified with sodium carbonate, filtered, and dried^4^.

### 2-(4-oxo-2-phenylquinazolin-3(4H)-yl) ethyl pentanoate (8a)

Buff powder; yield 83%, m.p 165 °C; IR (KBr) v/cm^−1^: 3006 (CH-arom), 2920 (CH-aliph), 1650 (C═O); ^1^H NMR (DMSO-d_6_) δ ppm: 0.80 (t , 3H , CH_3_), 1.19–1.59 (m, 4H, 2CH_2_), 1.92 (t, 2H, CH_2_), 3.50 (t, 2H, CH_2_N), 4.32 (t, 2H, CH_2_O), 7.57–7.98 (m, 9H, Ar–H); ^13^C NMR (DMSO-d_6_) δ ppm:14.49, 22.95, 25.50, 32.80, 39.90, 61.95, 121.45, 123.90, 127.65, 128.30, 128.50, 129.49, 130.32, 131.20, 134.77, 155.00, 165.37, 168.14, 170.00 ; Anal. Calculated for C_21_H_22_N_2_O_3_ (350.41); C, 71.98%, H , 6.33% , N , 7.99% ; Founded ; C , 72.18% , H, 6.43%, N, 8.39%.

### 2-(4-oxo-2-phenylquinazolin-3(4H)-yl) ethyl butyrate (8b)

Buff powder; yield 80%, m.p 156 °C; IR (KBr) v/cm^−1^: 3062 (CH-arom), 2958 (CH-aliph), 1615 (C═N), 1598 (C═O); ^1^H NMR (DMSO- d_6_) δ ppm: 0.86 (t, 3H, CH_3_), 1.59 (m, 2H, CH_2_ ), 2.01 (t, 2H, CH_2_), 3.48 (t, 2H, CH_2_N), 4.38 (t, 2H, CH_2_O), 7.24–7.62 (m, 9H, Ar–H); ^13^C NMR (DMSO-d_6_) δ ppm: 13.50, 18.40, 36.10, 38.70, 61.80, 120.00, 122.40, 126.10, 127.40, 128.70, 128.80, 128.90, 130.20, 133.50, 151.30, 161.50, 164.00, 173.10; Anal. Calculated for C_20_H_20_N_2_O_3_ (336.38); C , 71.41% , H , 5.99% , N , 8.33% ; Founded ; C , 71.40% , H , 6.19% , N , 7 .39% .

### General procedure for the synthesis of 2-(4-oxo-2-phenylquinazolin-3(4H)-yl) ethyl 2-ethylpentanoate (9a, b)

Compound **8a, b** (3.5 mmol) was dissolved in 20.0 mL THF. then CHIPA (0.6 mL, 6.0 mmol) was added at −96 °C, after that *n*-BuLi (0.4 mL, 6.0 mmol) was added drop wise with stirring then the reaction mixture was stirred for 60 minutes. Finally, alkyl halide (7.0 mmol) was added drop wise at −40 °C, then the reaction mixture was stirred overnight, the product was filtered off, dried under vacuum and weighed to give compounds **9a, b**^4,14^**.**

White powder ; yield 81%, m.p 161 °C ; IR (KBr) v/cm^−1^: 3066 (CH-arom), 2978 (CH-aliph), 1533(C═O); ^1^H NMR (DMSO- d_6_) δ ppm: 0.96 (t, 6H, 2CH_3_), 1.29-1.72 (m, 6H, 3CH_2_), 2.31 (m, 1H, CH), 3.28 (t, 2H, CH_2_N), 4.29 (t, 2H, CH_2_O), 7.40–7.72 (m, 9H, Ar–H); ^13^C NMR (DMSO-d_6_) δ ppm: 11.30, 14.10, 20.00,24.40, 32.50, 38.30, 46.60, 61.50, 120.90, 122.40, 126.10, 127.40, 128.70, 128.80, 130.20, 133.50, 151.30, 157.00, 164.00, 174.60; Anal.Calculated for C_23_H_26_N_2_O_3_ (378.46) ; C , 72.99%, H, 6.92%, N, 7.40% ; Founded; C, 73.09%, H, 7,12%, N, 7.8%.

### General procedure for the synthesis of 2-ethylpentanoic acid (10a, b)

A mixture of compound **9a, b** (0.6 g, 5.0 mmol) and methane sulphonic acid (few drops) in 20 .0 mL Dioxane was refluxed for 12 hours. Finally, the reaction mixture was poured into ice water, filtered and the target product was extracted with methylene chloride, dried with anhydrous sodium sulphate to give pale yellow liquid^14^**.**

Yield 88%; IR (KBr) v/cm^−1^: 3400 (OH), 2859 (CH-aliph); 1725 (CO); ^1^H NMR (DMSO- d_6_) δ ppm: 0.88 (t, 6H, 2CH_3_), 1.30–1.69 (m, 6H, 3CH_2_), 2.34 (m, 1H, CH), 11.22 (s, 1H, COOH); ^13^C NMR (DMSO-d_6_) δ ppm: 9.16, 13.10, 23.60, 30.70, 44.50, 55.82, 178.50; Anal. Calculated for C_7_H_14_O_2_ (130.18); C, 64.58%, H, 10.84%; Founded; C, 64.18%, H, 10.54%.

### General procedure for the synthesis of 2-ethyl-N-((R)-2-hydroxy-1-phenylethyl) pentanamide (11a, b)

Compound **10a, b** (0.6 g, 5.0 mmol) was added to (2.2 mL, 30.0 mmol) of thionyl chloride. The reaction mixture was refluxed for 4 hours and the excess of thionyl chloride was evaporated, and the acid chloride was dissolved in 10.0 mL THF. then the acid chloride was added drop wise to a solution of optically active (*R*)-phenyl glycinol (0.7 g, 5.0 mmol) in 10.0 mL THF, then the reaction mixture was refluxed for 6 h (TLC control), the diastereomeric amide **(11a, b)** was extracted with methylene chloride, washed with 1 N HCl then 1N NaOH, filtered and dried over sodium sulphate anhydrous^14^.

Yield 85–87% ; IR (KBr) v/cm^−1^: 3415 (NH), 3240 (OH), 1725 (CO); ^1^H NMR (DMSO- d_6_) δ ppm: 0.91 (t, 6H, 2CH_3_), 1.36–1.66 (m, 6H, 3CH_2_), 2.30 (m, 1H, CH), 3.59 (s, 1H, OH), 3.69 (d, 2H, CH_2_), 4.96 (t, 1H, CH), 7.25–7.80 (m, 5H, Ar–H), 8.0 (s,1H, NH); ^13^C NMR (DMSO-d_6_) δ ppm: 11.50, 14.70, 19.30, 21.90, 35.60, 46.50, 52.60, 69.80, 126.80, 127.00, 128.60, 139.20, 175.10; Anal. Calculated for C_15_H_23_NO_2_ (249.35); C, 72.25%, H, 9.30%, N, 5.62%; Founded; C, 72.85%, H, 9.1%, N, 5.52%.

### Separation of diastereomers using HPLC with silica gel column

The diastereomeric compounds **(7a, b and 11a, b)** were dissolved in methylene chloride and separated by HPLC using a silica gel column of 250×4.60 mm/Si 60–5 Mm, eluents: petroleum ether: ethyl acetate 8:2, flow rate =1 mL/min, detector: UV 254 nm^14^.

### Molecular docking and ADMET prediction

The exo-1,3-beta glucanase (PDB ID: 4m80) (https://www.rcsb.org/structure/4M80) and outer membrane protein A (PDB ID: 2ge4) (https://www.rcsb.org/structure/2GE4) target proteins were docked to the newly synthesized quinazolinone derivatives. Chemdraw Ultra 8.0 (https://en.freedownloadmanager.org/users-choice/Chemdraw_Ultra_8.0.) was used to create the 3D ligand molecules. To achieve more stable results, the ligand molecules and target proteins were then subjected to energy minimization before the docking technique was applied. The software Discovery Studio® Visualizer 2016 (https://discover.3ds.com/discovery-studio-visualizer-download) was used to depict the interactions^15^. Additionally, the SwissADME online tool (http://www.swissadme.ch/) was used to forecast the ADMET values, which are critical for drug design and the toxicity results were confirmed invitro using WI-38 and WISH normal cell lines^16^.

### Antimicrobial activities

The evaluated bacterial and fungal strains were tested for susceptibility to the synthesized compounds by measuring the diameter of the inhibitory zone using the agar well diffusion technique. The compounds were synthesized and then dissolved in solutions containing 0.1% DMSO at a concentration of 60 mg/mL. To compare the effectiveness of the bacterial and fungal strains, respectively, Ampicillin (5 µg) and Clotrimazole (10 µg) were used as positive controls, and 0.1% DMSO was used as a negative control. The bacterial and fungal strains were sub-cultured in nutrient broth medium for an entire day before being concentrated to 10^6^ CFU/mL at 630 nm. A 100 µL aliquot of each broth culture was evenly seeded throughout the nutrient agar medium using a sterile disposable plastic rod. On the surface of the nutritional agar medium, 9 mm wells were successfully made using a sterile cork porer, and 50 µL of each compound was then added^17^. The % activity index for the complex was calculated by the formula as follow:

$$\% {\text{ Activity Index}} = \frac{{{\text{Zone of inhibition by test compound }}\left( {{\text{diametre}}} \right)}}{{{\text{Zone of inhibition by standard }}\left( {{\text{diametre}}} \right)}} \times 100$$

### Statistical analysis

Data have been analyzed using the GraphPad Prism software (San Diego, CA, USA) (GraphPad Prism 6, https:// www.graph pad. com/ scientific- software/ prism/). The experimental data are expressed as mean ± SE; n = 3. The significance of the difference was analyzed using the one-way ANOVA. The acceptable significance was recorded when the *p-*value was < 0.05.

## Results and discussion

### Chemistry

**Figure 3:**
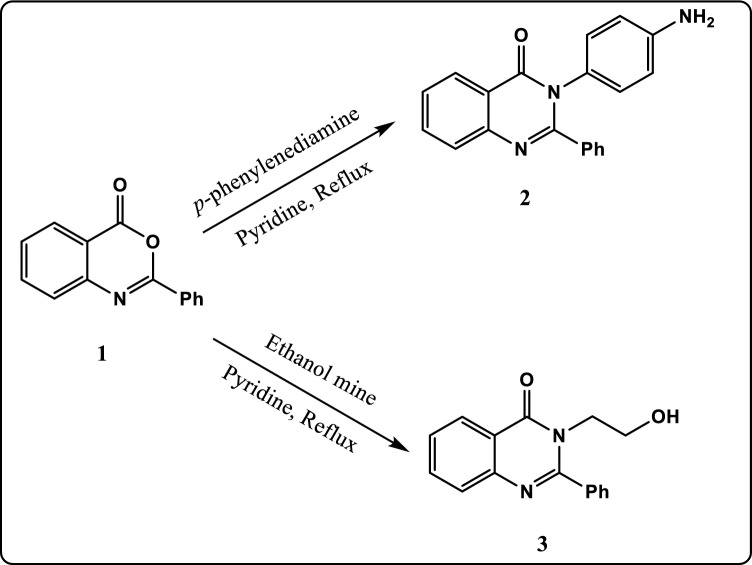
Synthesis pathway of compounds **2, 3.**

**Figure 4:**
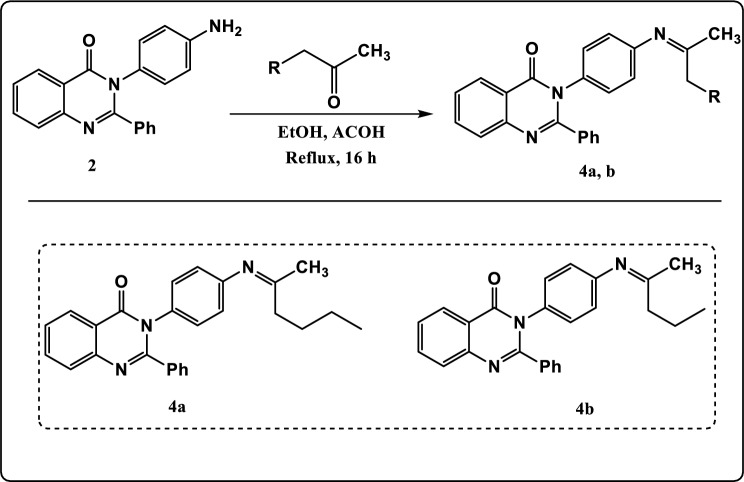
Synthesis pathway of compounds **4a, b.**

**Figure 5:**
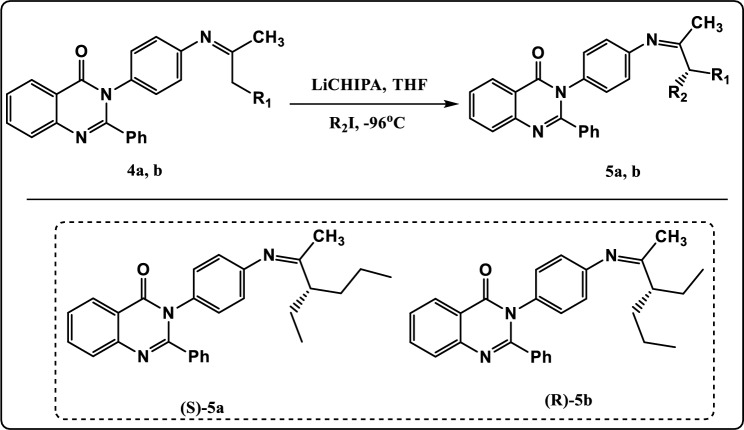
Synthesis pathway of compounds **5a, b.**

**Figure 6:**
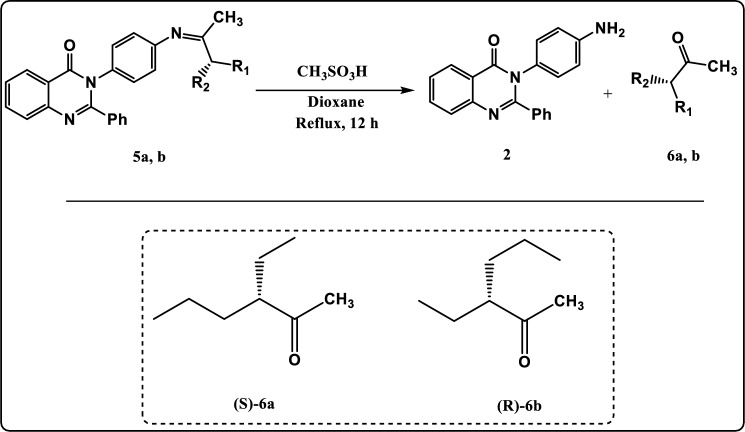
Synthesis pathway of compounds **6a, b.**

**Figure 7:**
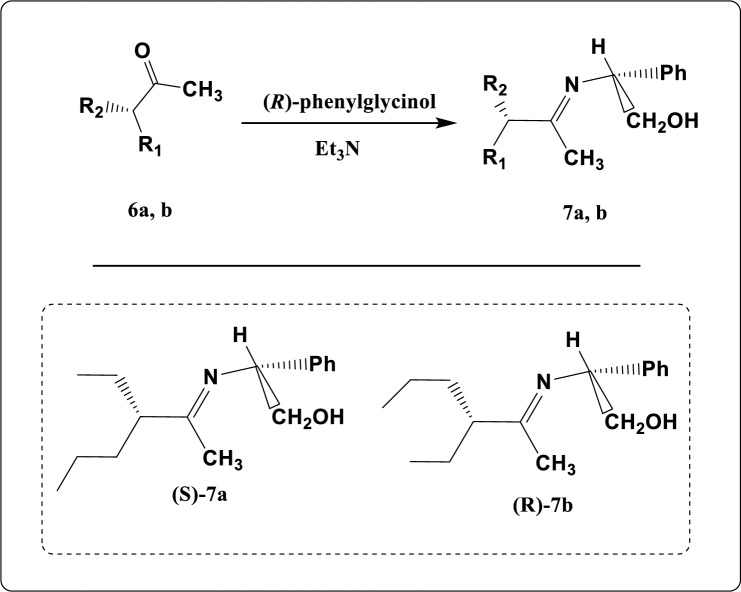
Synthesis pathway of compound **7a, b.**

**Figure 8:**
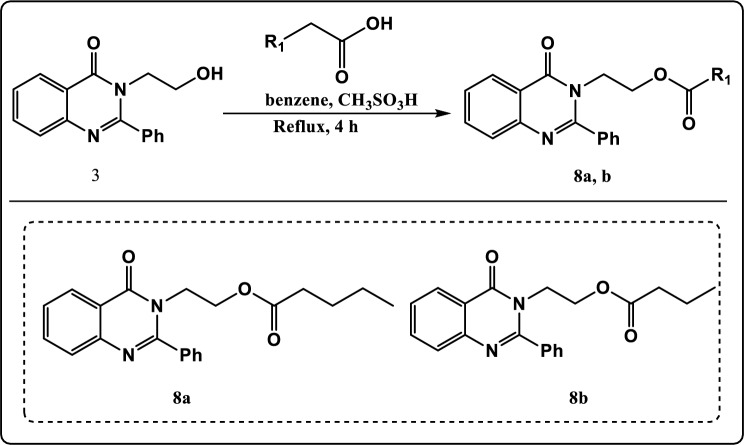
Synthesis pathway of compound **8a, b.**

**Figure 9:**
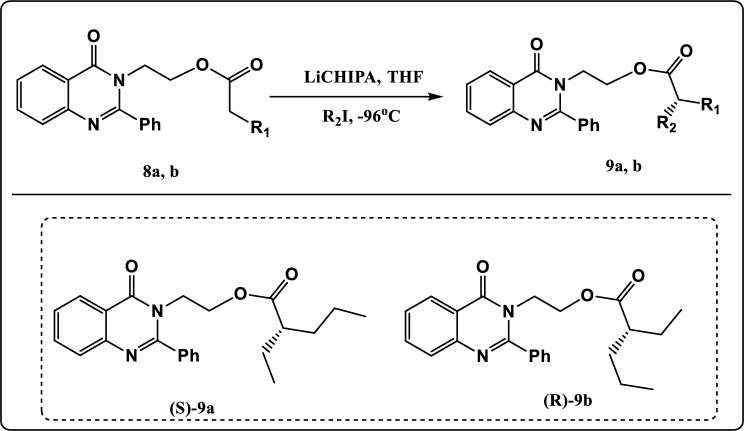
Synthesis pathway of compound **9a, b.**

**Figure 10:**
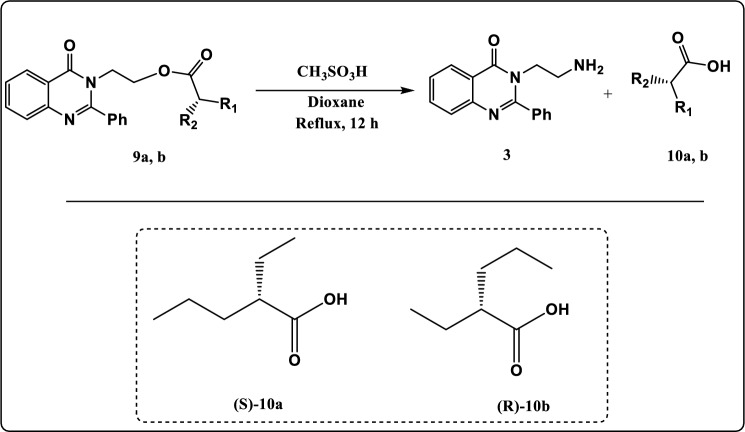
Synthesis pathway of compound **10 a, b.**

**Figure 11:**
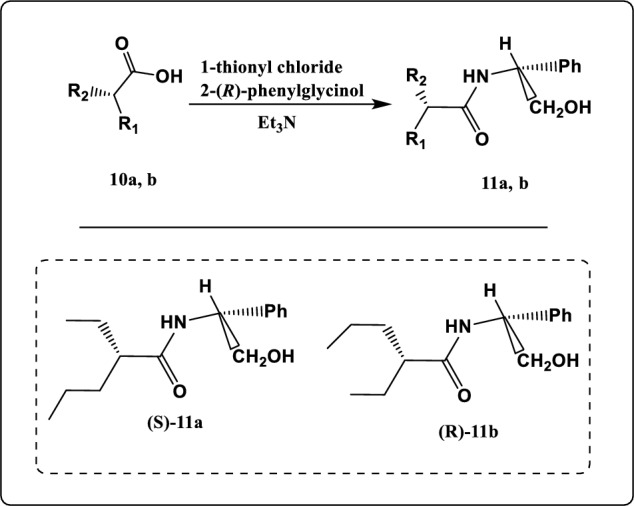
Synthesis pathway of compound **11a, b.**

The general approach for the synthesis of the designed compounds was illustrated in Figs. 3, 4, 5, 6, 7, 8, 9, 10 and 11. As shown in Fig. 3, the reaction of the starting compound, 2-phenyl-4-H-benzo[d][1,3]oxazin-4-one (**1**) with *p*-phenylene diamine or ethanol amine in presence of pyridine lead to formation of 3-(4-amino phenyl)-2-phenylquinazolin-4(3H)-one (**2**) and 3-(2-hydroxyethyl)-2-phenylquinazolin-4(3H)-one (**3**) respectively. The chemical structure of compounds **2** and **3** was established according to their elemental analysis and spectral data as described previously by Vijayakumar^12^ and Yang^13^ respectively.

As described in Fig. 4**,** the chemical modification of compound **2** with 2-pentanone or 2-hexanone in acidic medium led to formation of *Z*-3-(4-hexan-2-ylideneamino) phenyl)-2-phenylquinazolin-4-(3H)-one (**4a**) and *Z*-3-(4-pentan-2-ylideneamino) phenyl)-2-phenylquinazolin-4-(3H)-one (**4b**) respectively whose structures were determined on the basis of their elemental analysis and spectral data. The FT-IR spectrum of compound **4a** revealed absorption band at 1534 cm^−1^ characteristic for C=N group, 1642 cm^−1^ identical to C=O group, 2923 cm^−1^ due to CH-Aliphatic, 3090 cm^−1^ due to CH-Aromatic and a complete loss of NH_2_ stretching. The ^1^H NMR spectrum exhibited triplet signal at *δ* 0.80 ppm characteristic of methyl protons (CH_3_)_,_ multiplet signals at *δ* 1.15–1.30 ppm characteristic of methylene protons (2CH_2_), triplet signal at *δ* 1.40 ppm characteristic of methylene protons (CH_2_) and singlet signal at *δ* 2.01 ppm characteristic of methyl proton (CH_3_). The ^13^C NMR spectrum of compound **4a** showed signals at *δ* 170.65 and *δ* 165.19 which can be assigned to 2C=N groups. The FT-IR spectrum of compound **4b** showed absorption band at 1587 cm^−1^ due to C=N group, 1682 cm^−1^ for C=O group and a complete loss of NH_2_ stretching. The ^1^H NMR spectrum exhibited triplet signal at *δ* 0.80 ppm characteristic of methyl protons (CH_3_)_,_ multiplet signals at *δ* 1.12 ppm characteristic of methylene protons (CH_2_)_,_ triplet signal at *δ* 1.29 ppm characteristic of methylene protons (CH_2_) and singlet signal at *δ* 2.02 ppm characteristic of methyl proton (CH_3_). The ^13^C NMR spectrum exhibited signals at *δ* 170.70 and *δ* 165.19 which can be assigned to 2C=N groups.

Moreover, the deprotonation of compounds **4a, b** via lithium-cyclohexyl isopropyl amine (LiCHIPA) at −96 °C followed by α-alkylation with different alkyl halide led to formation of compounds **5a, b**. (Fig. 5). The FT-IR spectrum of compound **5a** revealed absorption band at 1596 cm^−1^characteristic for C=N group, 1636 cm^−1^ identical to C=O group, 2855 cm^−1^ due to CH-Aliphatic, 3024 cm^−1^ due to CH-Aromatic. The ^1^H NMR spectrum exhibited triplet signals at *δ* 0.89 ppm characteristic of methyl protons (2CH_3_)_,_ multiplet signals at *δ* 1.13–1.27 ppm characteristic of methylene protons (3CH_2_)_,_ multiplet signal at *δ* 1.30 ppm characteristic of CH proton_,_ and singlet signal at *δ* 1.98 ppm characteristic of methyl proton (CH_3_). The ^13^C NMR spectrum of compound **5a** showed signals at *δ* 171.90 and *δ* 164.90 which can be assigned to 2C=N groups.

In addition, the complete hydrolysis of 3-(4-(3-ethylhexan-2-ylideneamino) phenyl)-2-phenylquinazolin-4(3H)-one (**5a, b**) via methane sulphonic acid lead to formation of both 3-ethylhexan-2-one (**6a, b**) and 3-(4-aminophenyl)-2-phenylquinazolin-4(3H)-one (**2**) (which can be reused again) as described in Fig. 6. The chemical structure of compounds **6a, b** was confirmed by elemental analysis and spectral data. The FT-IR spectrum of compound **6a** revealed an absorption band at 1739 cm^−1^ identical to C=O group, 2922 cm^−1^ due to CH-Aliphatic and a complete loss of both C=N stretching and CH-aromatic stretching which demonstrated the completion of the hydrolysis process**.** The ^1^H NMR spectrum exhibited triplet signals at *δ* 0.81 ppm characteristic of methyl protons (2CH_3_)_,_ multiplet signals at *δ* 1.19–1.41 ppm characteristic of methylene protons (3CH_2_)_,_ singlet signal at *δ* 1.95 ppm characteristic of methyl protons (CH_3_)_,_ multiplet signal at *δ* 2.19 ppm characteristic of CH proton. The ^13^C NMR spectrum of compound **6a** showed signal at *δ* 212.00 which can be assigned to C=O group.

Furthermore, (2R)-2-(3-ethylhexan-2-ylideneamino)-2-phenylethanol (**7a, b)** was obtained via the reaction of compound **6a, b** with ethanolic solution of optically active (*R*)-phenyl glycinol as described in Fig. 7. The chemical structure of these compounds was established according to their elemental analysis as well as spectral data.

Additionally, as illustrated in Fig. 8, the chemical modification of compound **3** with pentanoic acid or butyric acid in acidic medium using dean stark trap led to formation of 2-(4-oxo-2-phenylquinazolin-3(4H)-yl)ethyl pentanoate (**8a**) and 2-(4-oxo-2-phenylquinazolin-3(4H)-yl)ethyl butyrate (**8b**) respectively whose structures were determined on the basis of their elemental analysis and spectral data. The FT-IR spectrum of compound **8a** revealed absorption band at 1650 cm^−1^ identical to C=O group, 2920 cm^−1^ due to CH-Aliphatic, 3006 cm^−1^ due to CH-Aromatic and a complete loss of OH stretching. The ^1^H NMR spectrum exhibited triplet signal at *δ* 0.80 ppm characteristic of methyl protons (CH_3_)_,_ multiplet signals at *δ* 1.19–1.59 ppm characteristic of methylene protons (2CH_2_)_,_ triplet signal at *δ* 1.92 ppm characteristic of methylene protons (CH_2_), triplet signal at *δ* 3.50 ppm characteristic of methylene protons (CH_2_N), and triplet signal at *δ* 4.32 ppm characteristic of methylene protons (CH_2_O). The ^13^C NMR spectrum of compound **8a** showed signals at *δ* 170.00 and *δ* 165.37 which can be assigned to 2C=O groups. The FT-IR spectrum of compound **8b** revealed absorption band at 1615 cm^−1^ identical to C=O group, 2958 cm^−1^ due to CH-Aliphatic, 3062 cm^−1^ due to CH-Aromatic and a complete loss of OH stretching. The ^1^H NMR spectrum exhibited triplet signal at *δ* 0.86 ppm characteristic of methyl protons (CH_3_)_,_ multiplet signals at *δ* 1.59 ppm characteristic of methylene protons (CH_2_)_,_ triplet signal at *δ* 2.01 ppm characteristic of methylene protons (CH_2_), triplet signal at *δ* 3.48 ppm characteristic of methylene protons (CH_2_N), and triplet signal at *δ* 4.38 ppm characteristic of methylene protons (CH_2_O). The ^13^C NMR spectrum of compound **8b** showed signals at *δ* 173.10 and *δ* 164.00 which can be assigned to 2C=O groups.

The asymmetric alkylation of compounds** 8a, b** at −96 °C led to formation of compounds **9a, b**. (Fig. 9). The FT-IR spectrum of compound **9a** showed an absorption band at 1533 cm^−1^ identical to C=O group, 2978 cm^−1^ due to CH-Aliphatic, 3066 cm^−1^ due to CH-Aromatic. The ^1^H NMR spectrum exhibited triplet signal at *δ* 0.96 ppm characteristic of methyl protons (2CH_3_)_,_ multiplet signals at *δ* 1.29–1.72 ppm characteristic of methylene protons (3CH_2_)_,_ multiplet signal at *δ* 2.31 ppm characteristic of CH proton_,_ triplet signal at *δ* 3.28 ppm characteristic of methylene protons (CH_2_N), and triplet signal at *δ* 4.29 ppm characteristic of methylene protons (CH_2_O). The ^13^C NMR spectrum of compound **9a** showed signals at *δ* 174.60 and *δ* 157.00 which can be assigned to 2C=O groups.

As illustrated in Fig. 10, the hydrolysis of compounds **9a, b** in acidic medium gave (*S*)-2-ethylpentanoic acid (**10a**) and (*R*)-2-ethylpentanoic acid compounds (**10b)** respectively. The FT-IR spectrum of compound **10a** showed an absorption band at 1725 cm^−1^ identical to C=O group, 2859 cm^−1^ due to CH-Aliphatic, 3400 cm^−1^ due to OH. The ^1^H NMR spectrum exhibited triplet signal at *δ* 0.88 ppm characteristic of methyl protons (2CH_3_)_,_ multiplet signals at *δ* 1.30–1.69 ppm characteristic of methylene protons (3CH_2_)_,_ multiplet signal at *δ* 2.34 ppm characteristic of CH proton, and singlet signal at *δ* 11.22 ppm characteristic of carboxylic proton (COOH). The ^13^C NMR spectrum of compound **10a** showed signal at *δ* 178.50 which can be assigned to COOH group.

As illustrated in Fig. 11, The chemical reaction of compounds **10a, b** with thionyl chloride led to formation of 2-ethylpentanoyl chloride which on reaction with optically active (*R*)-phenyl glycinol led to formation of 2-ethyl-N-((R)-2-hydroxy-1-phenylethyl) pentanamide (**11a, b).** The structure of these compounds was established according to their elemental analysis and spectral data.

Furthermore, compounds **7a, b** and** 11 a, b** were separated via HPLC using silica gel column and give high values of enantiomeric ratio indicating that compounds **2**, **3** enhanced the steric hindrance during asymmetric alkylation process as tabulated in Table 1.

**Table 1:** Enantiomeric values of compounds **7a, b and 11 a, b**

Entry	Temperature^a^ (°C)	Yield^b^ (%)	Configuration^c^	e.r.^d^
7a	− 96	86	*S*	91:9
7b	− 96	84	*R*	6:94
11a	− 96	87	*S*	92:8
11b	− 96	85	*R*	6:94

Reaction conditions: ^a^deprotonation temperature during asymmetric alkylation. ^b^Yield of isolated products. ^c^configuration of the isolated products. ^d^The enantiomeric ratio (e.r.) was determined via HPLC analysis on silica gel column.

### In-silico and ADMET pharmacokinetics studies

The newly synthesized ligand molecules and the target proteins were docked together to learn more about the chemical interactions and binding scores^18^. Here, exo-1,3-beta-glucanase and outer membrane protein A (OMPA) are recognized as interesting therapeutic targets for the development of antibacterial and antifungal drugs. To understand the ligand compounds’ mode of action as antibacterial agents in the current work, the docking method utilized OMPA protein. The findings revealed that compound **6a** had the best binding affinity against the target OMPA protein (ΔG = −8.23 kcal mol^−1^) and the most promising bacterial inhibitory effect among all other newly synthesized compounds compared with the reference ampicillin FDA approved antibacterial drug that gave binding energy equal to −10.05 kcal mol^−1^. Compound **6a** bonded with the OMPA protein residues PHE353, LYS361, LEU352, ARG447, ARG405, ARG448, ILE351, GLU444 via hydrogen bonding and electrostatic interactions compared with the ampicillin reference drug that bonded to the OMPA target protein residues GLU444, PHE353, GLY356, ARG405, ARG447, THR355, LEU401, LEU352 by hydrogen bonding and electrostatic interactions.

**Figure 12:**
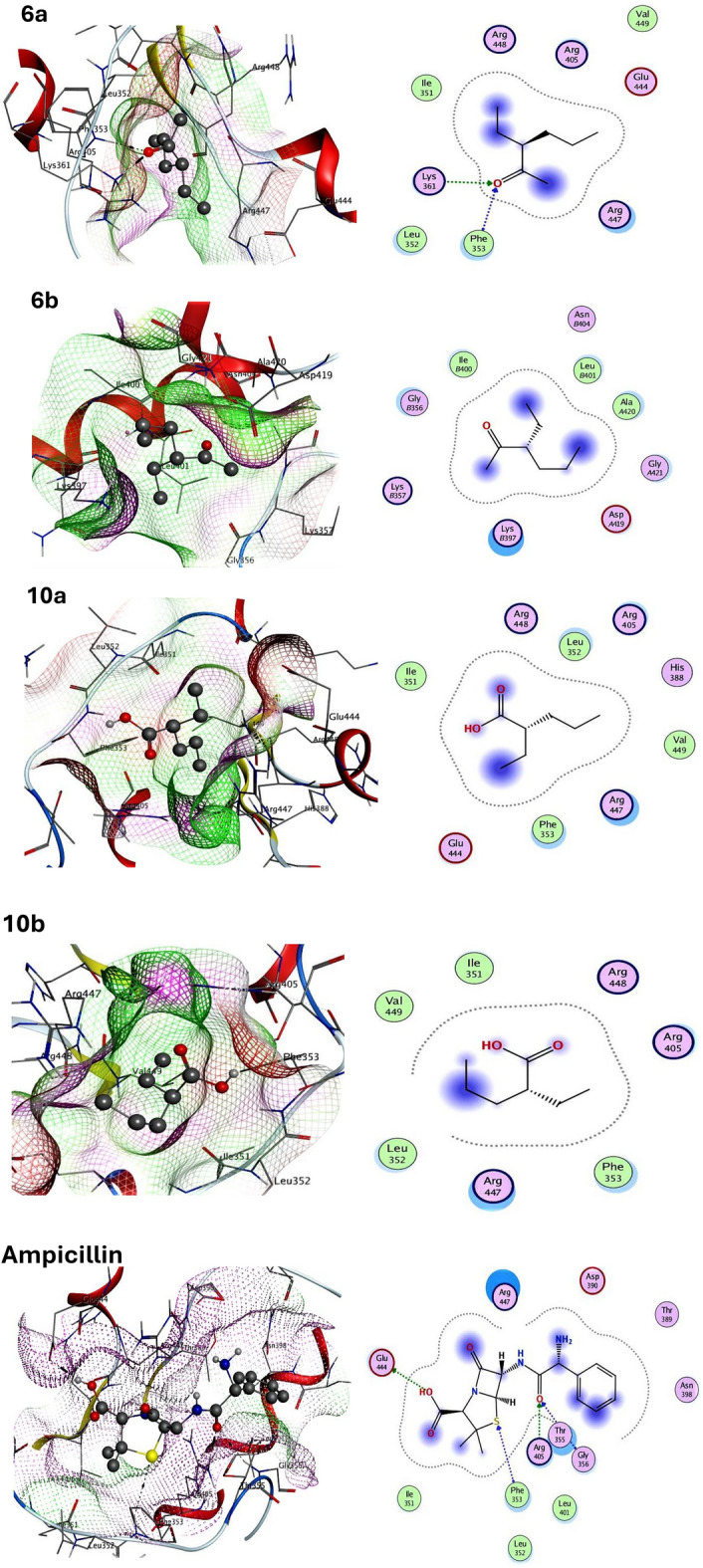
Molecular docking interactions of all synthesized compounds and reference drug with OMPA protein, 3D-(Left side) and 2D (Right side).

**Figure 13:**
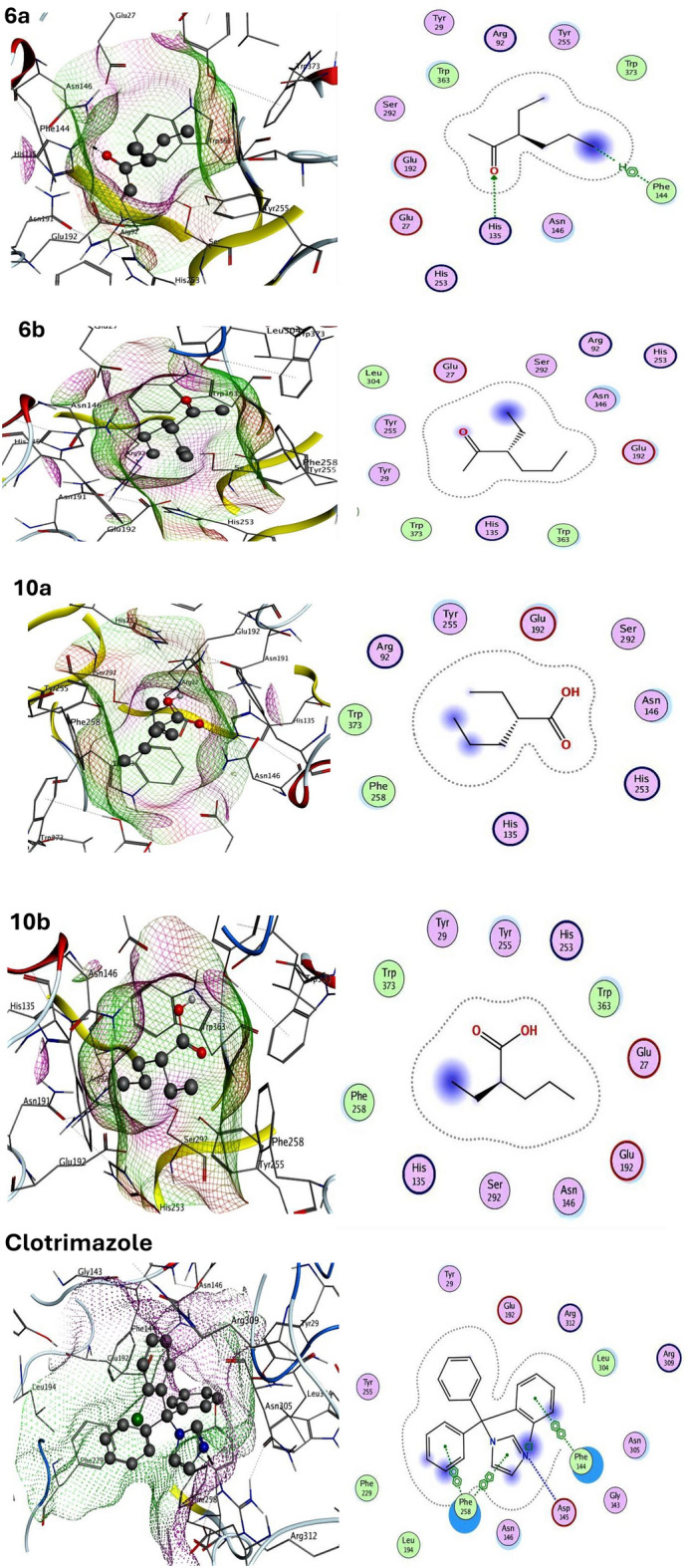
Molecular docking interactions of all synthesized compounds and reference drug with exo-1,3-beta-glucanase protein, 3D-(Left side) and 2D (Right side).

Moreover, to investigate the mechanism of action of the synthesized compounds as antifungal medicines, exo-1,3-beta-glucanase target protein was used in the docking simulation. The findings revealed that compound **6a** had the strongest binding affinity for the target exo-1,3-beta-glucanase protein (ΔG = −9.25 kcal mol^−1^) and the most promoting inhibitory activity of fungi compared with the clotrimazole reference FDA antifungal drug that gave binding energy equal to −9.89 kcal mol^−1^. Compound **6a** bonded to exo-1,3-beta-glucanase with hydrogen bonding, π and electrostatic interactions to the residues TRP363, TYR255, PHE144, ASN146, HIS135, GLU192, TRP373 compared with reference clotrimazole drug that bonded also with hydrogen bonding, π, and electrostatic interactions to the exo-1,3-beta-glucanase target protein residues ASP145, PHE258, PHE144, ASN146, ASN305. Thus, compound **6a** elucidated dual antibacterial and antifungal potent effects, according to the results elucidated in Table 2. Figures 12 and 13 showed the 2D and 3D molecular interactions network of newly synthesized compounds and reference drugs with the target proteins.

**Table 2:** Calculated docking scores (kcal/mol) of all synthesized compounds with the target proteins.

Compounds	OMPA		Exo-1,3-beta-glucanase	
	Docking Score (AGbind)	Docked complex (amino acid-ligand) interactions	DockingScore (AGbind)	Docked complex (amino acid-ligand) interactions
6a	− 8.23	H-Acceptor	− 9.25	H-Acceptor
		PHE353		HIS135
		LYS361		H-π interaction
		Electrostatic interactions		PHE144
				Electrostatic interactions
		LEU352		
		ARG447		TRP363
		ARG405		TYR255
		ARG448		ASN146
		ILE351		GLU192
		GLU444		TRP373
6b	− 4.65	Electrostatic interactions	− 4.42	Electrostatic interactions
		ILE400		TYR255
		GLY356		ASN146
		LYS397		GLU192
		LEU401		TYR29
		ALA420		TRP363
		GLY421		
10a	− 3.49	Electrostatic interactions	− 4.25	Electrostatic interactions
		PHE353		TYR255
		ARG447		GLU192
		LEU352		PHE258
		ARG405		ASN146
10b	− 4.01	Electrostatic interactions	− 4.93	Electrostatic interactions
		ARG447		ASN146
		PHE353		GLU192
		LEU352		PHE258
		ARG405		TYR255
				TRP363
Clotrimazole (Reference Fungicidal)	− 10.05	H-Donor		–
		GLU444		
		H-Acceptor		
		PHE353		
		GLY356		
		ARG405		
		Electrostatic interactions		
		ARG447		
		THR355		
		LEU401		
		LEU352		
Clotrimazole (Reference Fungicidal)	–		− 9.89	H-Donor
				ASP145
				π–π interaction
				PHE258
				PHE144
				Electrostatic interactions
				ASN146
				ASN305

**Figure 14:**
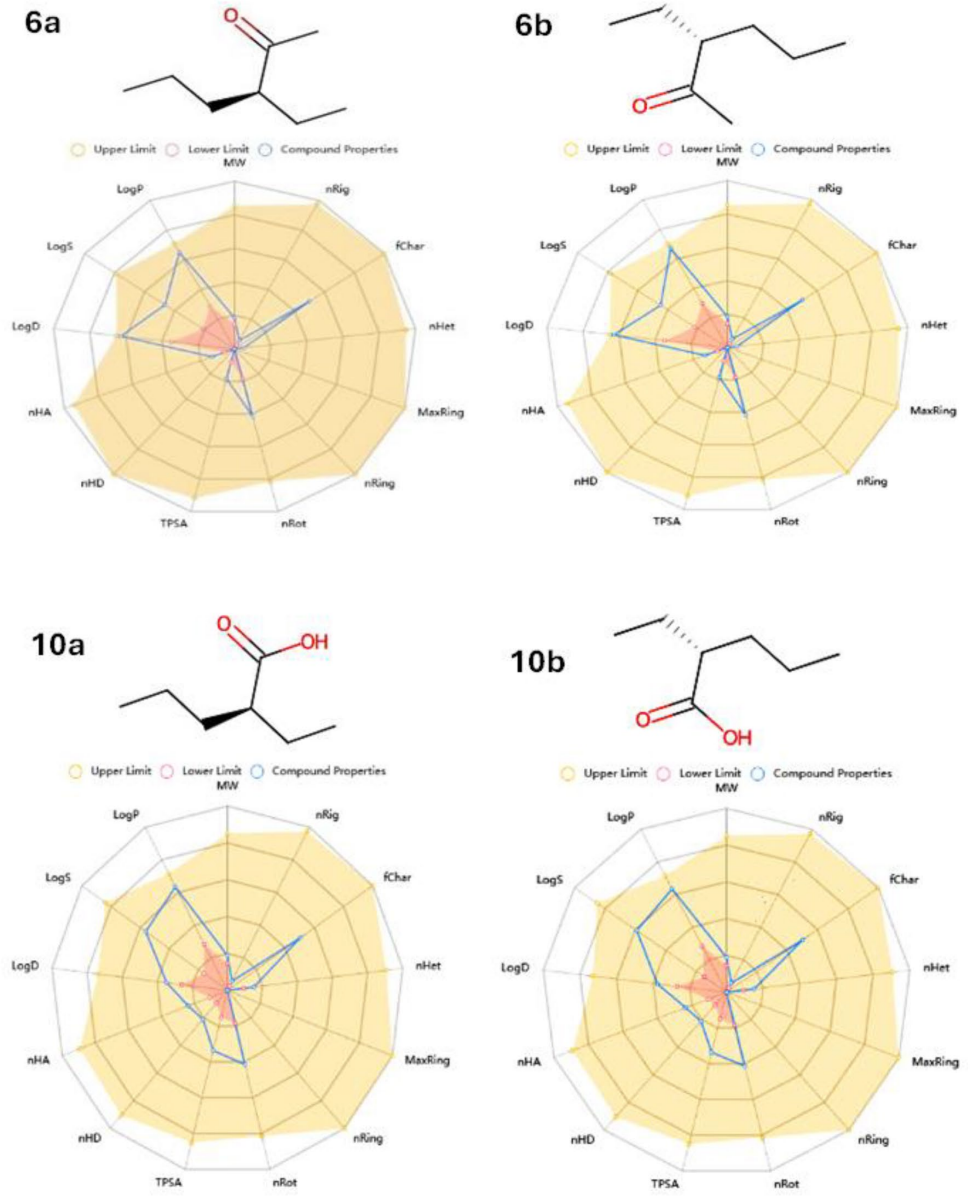
ADMET pharmacokinetics features for all synthesized compounds.

Furthermore, all synthetic compounds need to have their ADMET-determined pharmacokinetic characteristics. A radar map with 13 parameters was used to assess each compound’s bioavailability and physicochemical properties (Fig. 14). All the investigated compounds met all the criteria for good permeability, the Ro5 (no. of violations 0), and adequate oral bioavailability, as the TPSA range was 17.07–37.300 A^2^. They also demonstrated rotatable bonds with numbers ranging from 0 to 10, indicating flexibility. Because their HBA and HBD values were within the acceptable range, they had better solubility in cellular membranes. Log p values < 5 indicated good lipophilicity characteristics. Furthermore, according to the ADMET criterion, the compounds had higher Human Intestinal Absorption (% HIA) ratings, implying that the human gut could absorb them more effectively. Because the target chemicals do not cross the blood-brain barrier, they offer an excellent CNS safety profile. Finally, all AMES toxicity and carcinogenicity test results came back negative, demonstrating their safety (Table 3). Moreover, the in-vitro studies confirmed the AMES toxicity results and elucidated that the IC_50_ of all synthesized compounds (**6a**–**10b**) on WI-38 (normal lung cell line) was equal to 157.3 ± 1.23, 193.6 ± 2.1, 86.90 ± 2.2 and 117.3 ± 2.3 µM respectively. Also, all compounds were investigated their toxicity on WISH normal cell line and the IC_50_ values were equal to 180.5 ± 1.4, 156.0 ± 1.6, 160.2 ± 2.6 and 127.4 ± 2.7 µM respectively as shown in Figs. S13 and S14. Therefore, the insilico and invitro studies were inline with each other and revealing the compounds’ safety.

**Table 3:** ADMET properties of the synthesized compounds.

	Molecular weight (g/mol)	Blood–brain barrier (BBB)	Bioavailability score	%Human intestinal absorption (HIA +)	TPSA A^2^	Log p	HBA	HBD	N rotatable	AMES toxicity	Carcinogenicity
Acceptable ranges	< 500	NO	0.55–1.00	> 80% high < 30% low	< 140	< 5	2.0–20.0	0.0–6.0	< 10	Nontoxic	Noncarcinogenic
6a	128.120	No	0.55	95.34	17.07	2.57	1	0	4	Nontoxic	Noncarcinogenic
6b	128.215	No	0.55	94.36	17.07	2.77	1	0	4	Nontoxic	Noncarcinogenic
10a	130.100	No	0.85	96.48	37.30	2.42	2	1	4	Nontoxic	Noncarcinogenic
10b	130.100	No	0.85	97.44	37.30	2.46	2	1	4	Nontoxic	Noncarcinogenic

*TPSA* Total polar surface area, *BBB* Blood brain barrier, *HBA* Hydrogen bond acceptor, *HBD* Hydrogen bond donor, *Logp* Octanol/water partition coefficient.

### In-vitro antimicrobial activity

Antimicrobial resistance is influenced by outer membrane protein A (OMPA) and exo1,3 beta-glucanase. One of the main α-barrel porins expressed in bacterial outer membranes is called OMPA. This membrane has a variety of roles in the pathophysiology of bacteria, including resistance, induction of host cell death, and adhesion to host cells. Clinically, overexpression of the OMPA gene is linked to the onset of pneumonia and bacteremia, as well as patient death. A globular protein facing the periplasmic space, the C-terminal OmpA-like domain of outer membrane proteins interacts non-covalently with peptidoglycan to maintain cellular integrity and stability^19^. Furthermore, as the primary skeletal polysaccharides of fungal cell walls, β-1,3-glucanase catalyzes the hydrolytic cleavage of the β-1,3-D-glycosidic linkages in β-1,3-glucans. Therefore, inhibiting the OMPA and β-1,3-glucanases during the pathogenicity may appeared to be the primary role for treatment^20^.

**Figure 15:**
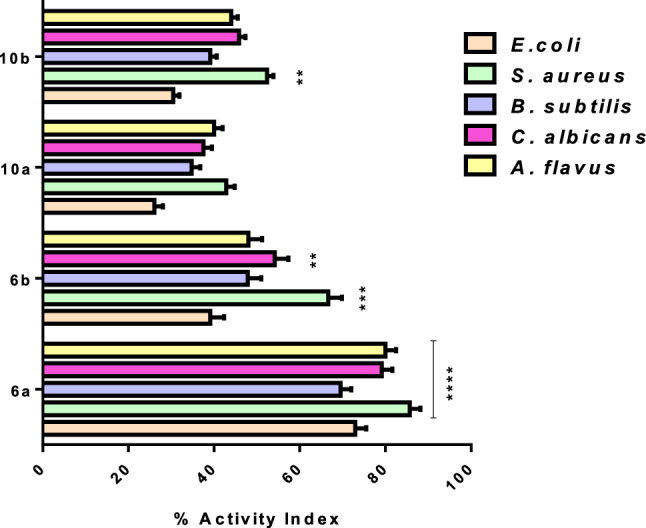
Activity index of all synthesized compounds against panel of bacterial and fungal strains. The experiment was done in triplicates and **p < 0.01, ***p < 0.001 and ****p < 0.0001 was significant.

According to the previous point of view, there was an urgent need to discover newly synthesized compounds that were effective against these target proteins without any adverse effects. Therefore, our finding elucidated the antimicrobial impact of the newly synthesized carbonyl compounds in-silico against OMPA and exo- β-1,3-glucanases and observed their safety pharmacokinetics profile using AMES examinations. Herein, the invitro disc diffusion antimicrobial method confirmed all the in-silico results as compound (*S*) 3-ethylhexan-2-one (**6a**) showed a highly bactericidal effect against all bacterial strains investigated *E. coli*, *S. aureus* and *B. subtilis* with activity index equal to 73±2.5, 85.71±2.72 and 69.56±1.8% respectively compared with ampicillin standard antibacterial drug. Further, (*S*) 3-ethylhexan-2-one (**6a**) demonstrated powerful significant *p*<0.0001 fungicidal effect against *C. albicans* and *A. flavus* with activity index values 79.18±2.01 and 80±1.9% respectively compared with clotrimazole standard antifungal drug (Table 4; Fig. 15).

**Table 4:** Inhibition diameter zones (mm) of all synthesized compounds.

Compounds	*E. coli*	*S. aureus*	*B. subtilis*	*C. albicans*	*A. flavus*
Diameter of inhibition zone (mm)					
6a	17 ± 1.1	18 ± 1.6	16 ± 1.42	19 ± 1.5	20 ± 1.4
6b	9 ± 0.9	14 ± 1.2	11 ± 1.0	13 ± 1.6	12 ± 1.0
10a	6 ± 0.7	9 ± 1.0	8 ± 0.5	9 ± 0.4	10 ± 0.4
10b	7 ± 0.6	11 ± 1.0	9 ± 0.3	11 ± 1. 2	11 ± 0.9
Ampicillin	23 ± 2.1	21 ± 1.5	23 ± 1.9	**–**	**–**
Clotrimazole	**–**	**–**	**–**	24 ± 2.1	25 ± 1.9

On the other hands, compound (*R*) 3-ethylhexan-2-one (**6b**) observed significant moderate impact against *S. aureus and C. albicans* with activity index 66.66±3.2% *p*<0.001 and 54.16±1.8 *p*<0.01 respectively and weak effect against *E. coli*, *B. subtilis*, and *A. flavus* with activity index 39.13±2.8, 47.82±1.6, and 48±3% respectively compared with ampicillin and clotrimazole reference drugs*.* Additionally, compound (S)-2-ethylpentanoic acid (**10a**) stated weak antimicrobial effect against all bacterial (*E. coli*, *S. aureus* and *B. subtilis*) and fungal (*C. albicans* and *A. flavus*) strains investigated as compound **10a** gave activity index equal to (26.08±2, 42.85±1.5, 34.78±1.1%), (37.50±2.2 and 40±2.5%) respectively. While compound (R)-2-ethylpentanoic acid (**10b**) had a significant *p*< 0.01 moderate activity index against *S. aureus* (52.38±1.7) and weak activity index against (*E. coli*, and *B. subtilis*) and fungal (*C. albicans* and *A. flavus*) strains investigated (30.43±1.4, 39.13±1%), (45.83±3.01 and 44±2.7%) respectively compared with ampicillin and clotrimazole reference drugs (Table 4; Fig. 15)*.* Ultimately, we would like to praise that this work was considered a new and modern breakthrough in the science of stereochemistry especially in the discovery of new drugs for antimicrobial treatment.

### Antimicrobial structure activity relationship (SAR)

**Figure 16:**
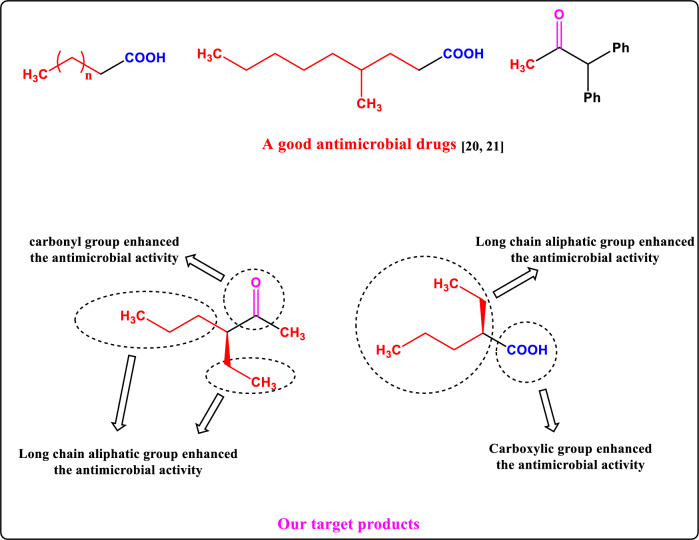
Structure activity relationship of the synthesized compounds.

As described in Fig. 16, the structure activity relationship demonstrated that the inhibitory effect on Gram-positive, gram negative bacterial and fungal species was due to that the compound could easily cross the peptidoglycan structure of the bacterial and fungal species and then destroy the cell wall structure of them, which leads to the leakage of intracellular substances and invading the bacterial and fungal cells play a bacteriostatic role. Additionally, Increasing the chain of long aliphatic will cause an increase in the corresponding activity^21,22^.

## Conclusion

In summary, an efficient method for the asymmetric synthesis of α-alkylated carbonyl compound was achieved. The structure of all compounds was confirmed via elemental analysis and different spectroscopic data and the enantioselectivity was determined via HPLC using silica gel column. In addition, the synthesized carbonyl compound was examined against different types of bacteria and fungi and compound **6a** gave positive potential antimicrobial activity results among the other synthesized compounds. Also, they obey Lipinski rule of 5.

### Limitations

- Synthesis of acid chloride via thionyl chloride requires full attention, a single fault while working can affect the eyes, lungs, or other body parts so the reaction must proceed in fume cupboard.
- Some chemical reactions proceeded at − 96 °C which need specific cooling device to reached to this temperature (we can use a mixture of methanol with liquid nitrogen as a cooling bath if the cooling device is not available)
- Most of reactions proceeded under nitrogen gas so, if the nitrogen system is not available, we can use nitrogen ballon at the top of the condenser.
- Cleaning, disinfection, and Sterilization for the removal of foreign material from objects is very important before testing the antimicrobial activity because inorganic and organic materials that remain on the surfaces of instruments interfere with the effectiveness of these processes.

### Recommendations

Our prospective recommendation is to complete studying these newly synthesized compounds on antibiotic microbial resistance. Also, we strongly recommend investigating their antitumor impact *invitro* and *in vivo* in further studies.

### Supplementary Information

Supplementary Information.

## Data Availability

The data that support the findings of this study are included within the article and supplementary file. The datasets generated and/or analyzed during the current study are available in: Macromolecule protein structure, can be deposited in the worldwide protein data bank repository, (https://www.rcsb.org/structure/4M80) and (https://www.rcsb.org/structure/2GE4).
