# Supplementary material for: Enantioselective synthesis, characterization, molecular docking simulation and ADMET profiling of α-alkylated carbonyl compounds as antimicrobial agents
Source: Sci Rep. 2024 May 21;14:11586. doi: 10.1038/s41598-024-61884-9 (PMC11109205; doi:10.1038/s41598-024-61884-9)
Supplement: Supplementary file 1 — Supplementary Information. [file 41598_2024_61884_MOESM1_ESM.pdf]

Enantioselective Synthesis, Characterization, Molecular docking simulation and ADMET profiling of α-alkylated carbonyl compounds as antimicrobial agents

Ahmed A. Noser [1]*, Mariam Ezzat [1], Shimaa G. Mahmoud[1], Adel I. Selim [1], and Maha M Salem [2]

[1] Chemistry Department, Faculty of Science, Tanta University, 31527, Egypt

[2] Biochemistry division, Chemistry Department, Faculty of Science, Tanta University, 31527, Egypt

Emails: **ahmed.nosir@science.tanta.edu.eg**

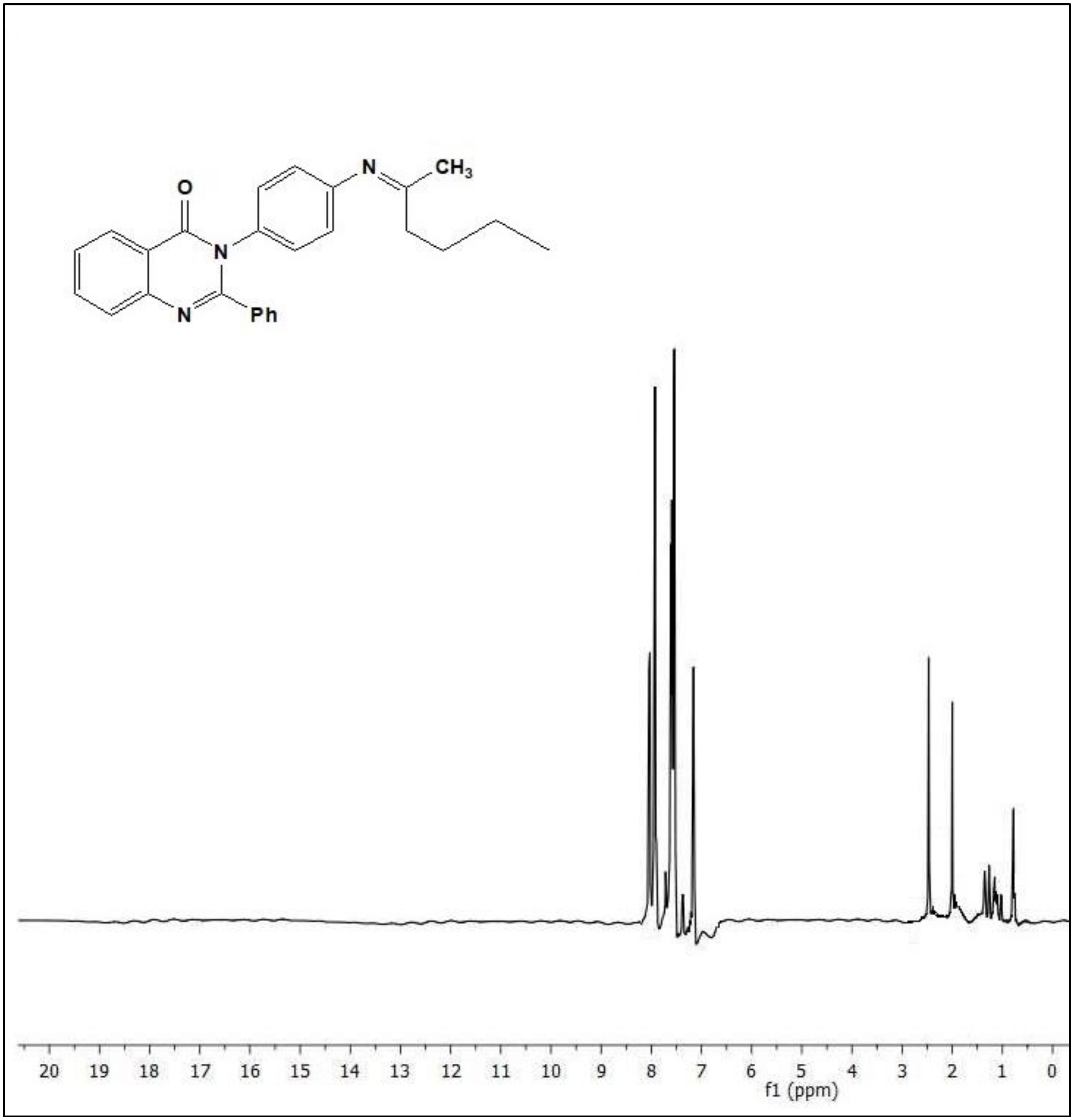

Figure S1: ^{1}HNMR spectra of compound 4a

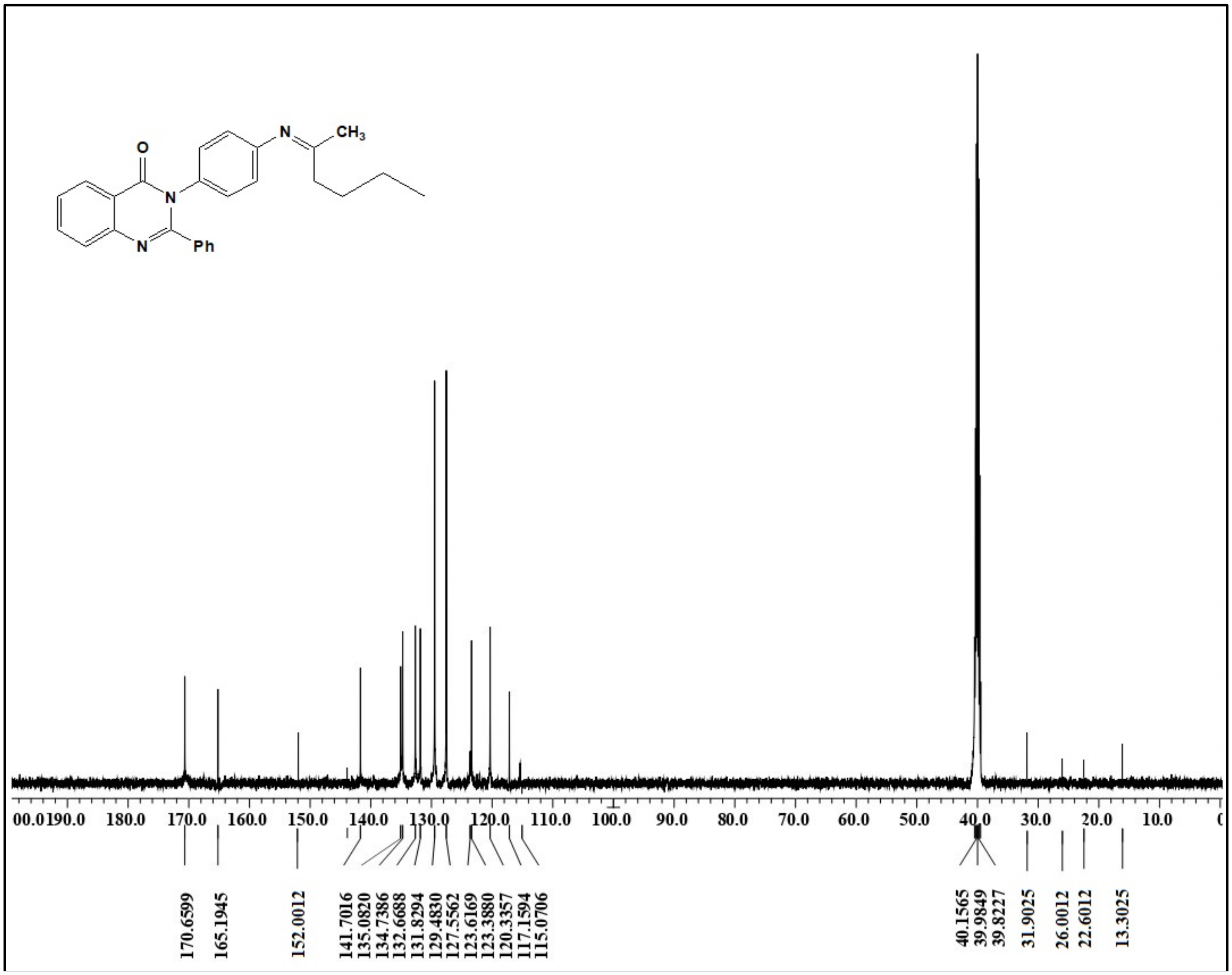

Figure S2: ^{13}CNMR spectra of compound 4a

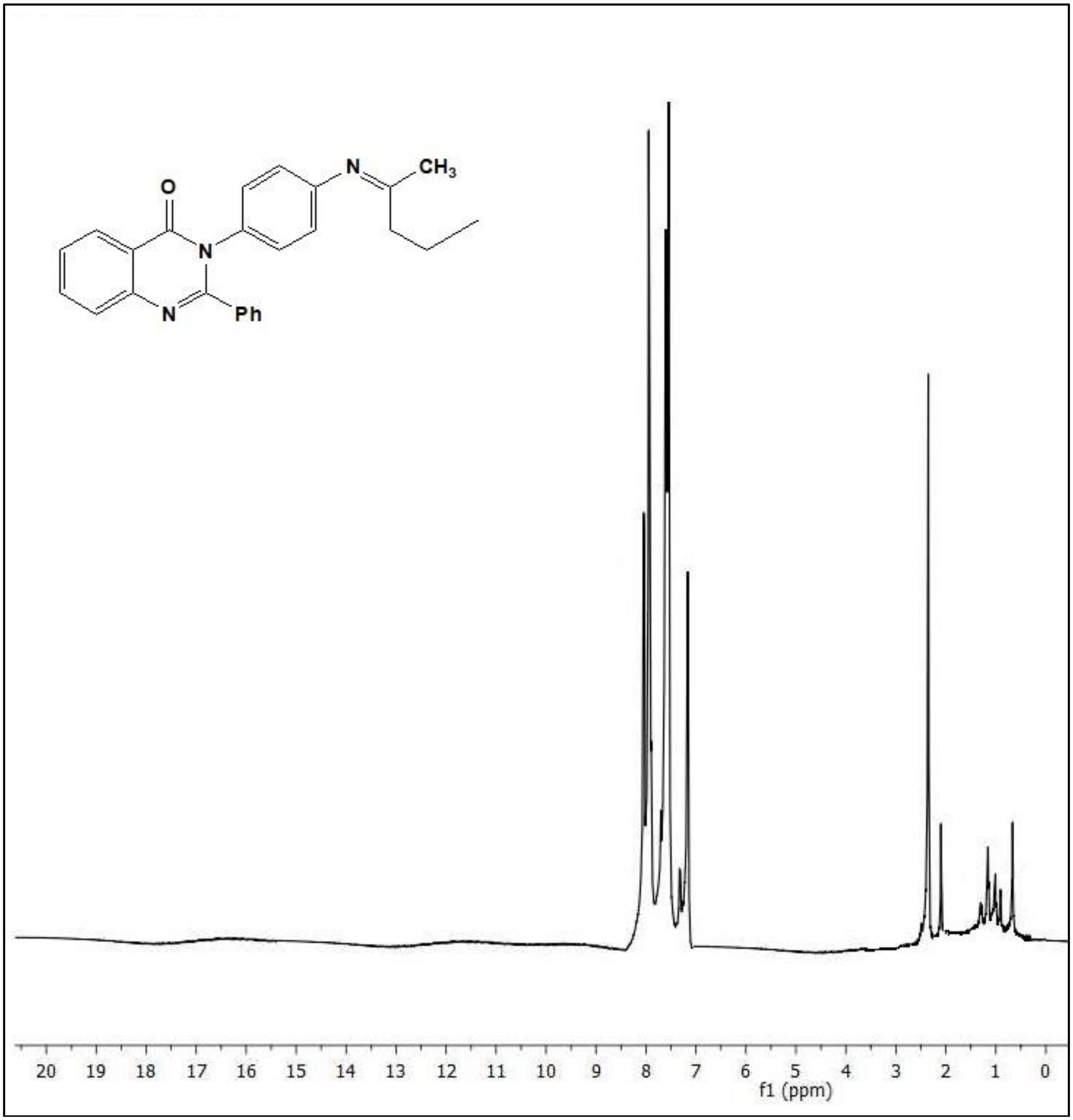

Figure S3: ^{1}HNMR spectra of compound 4b

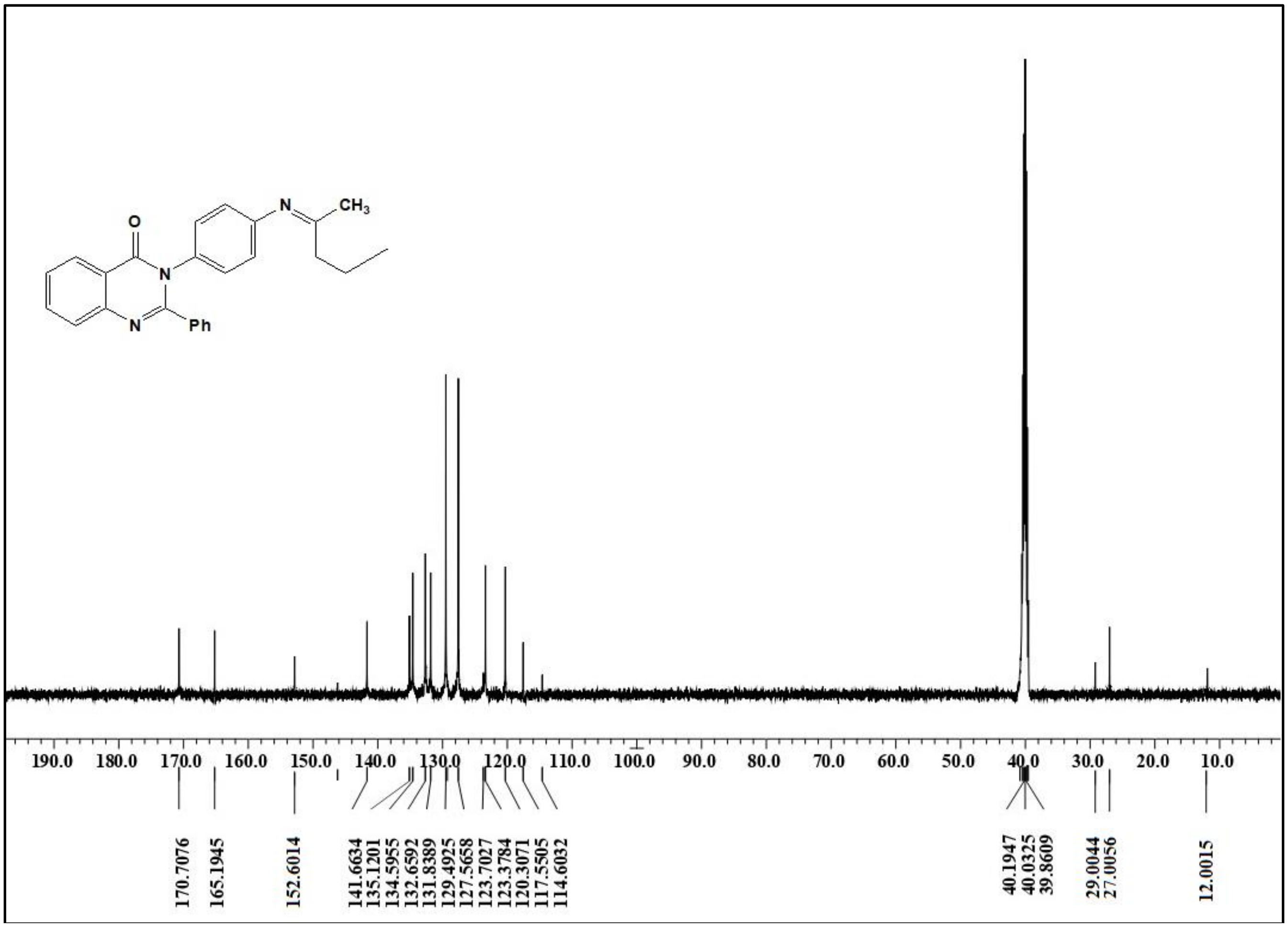

Figure S4: ^{13}CNMR spectra of compound 4b

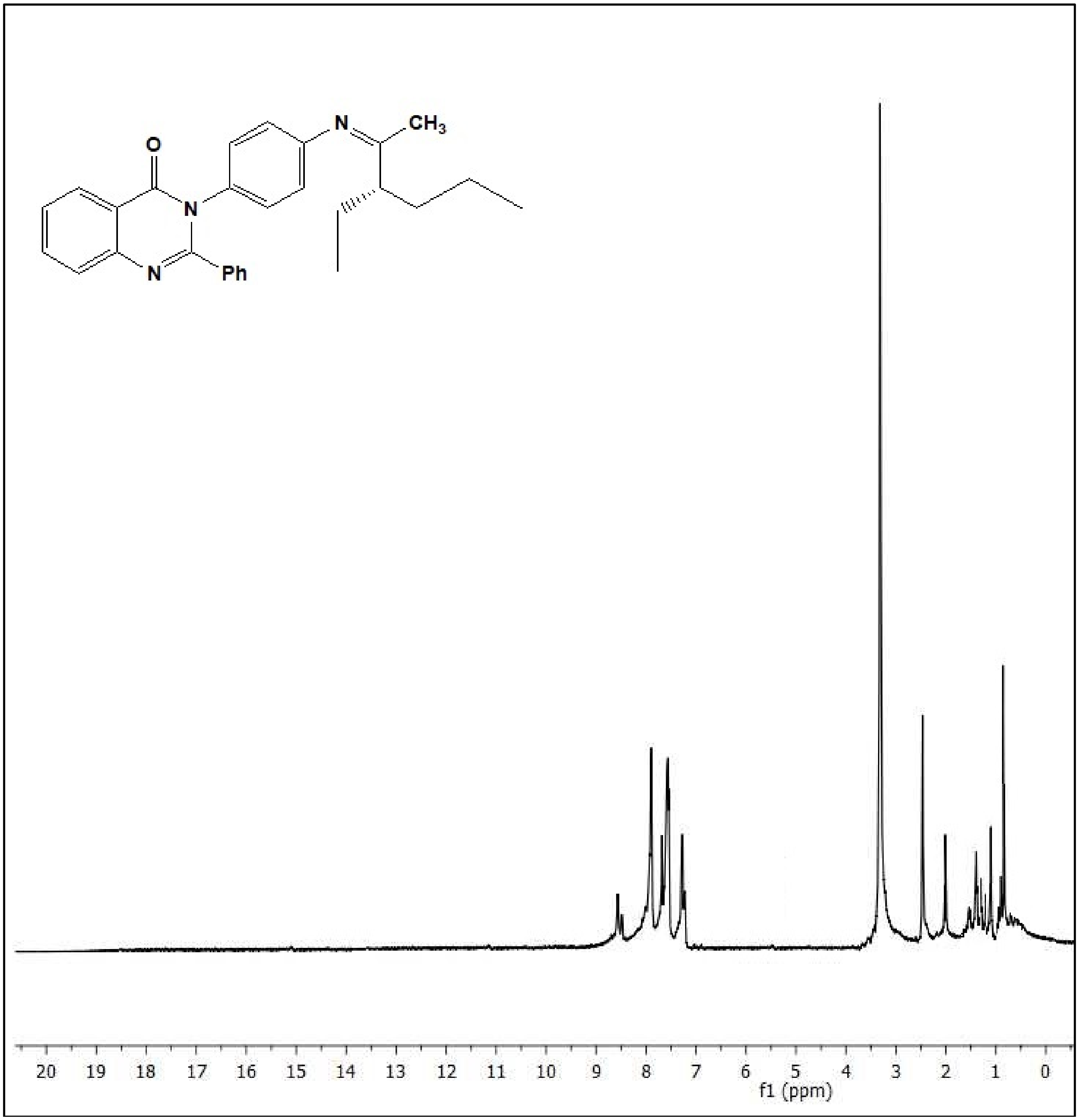

Figure S5: ^{1}HNMR spectra of compound 5a

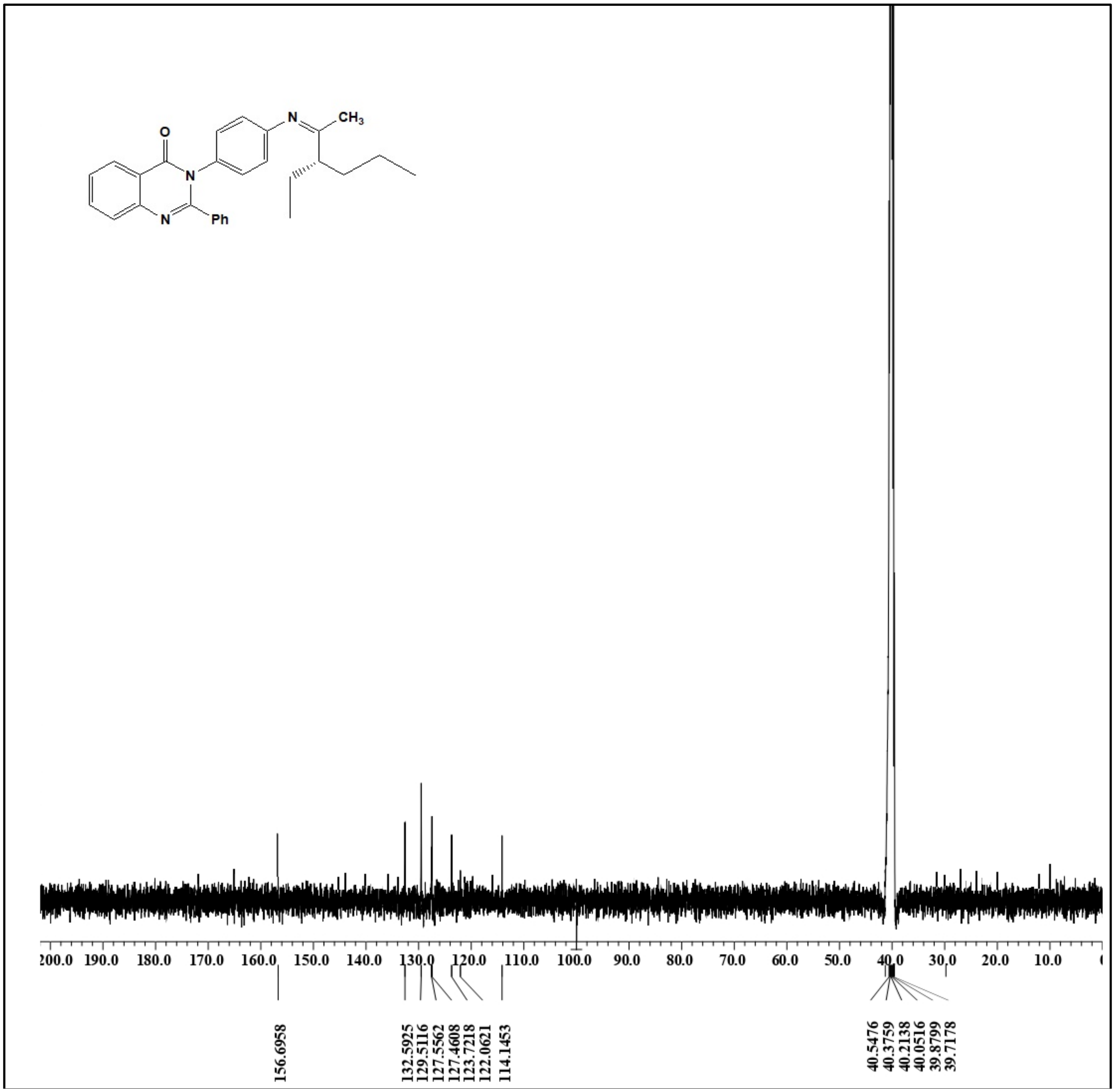

Figure S6: ^{13}CNMR spectra of compound 5a

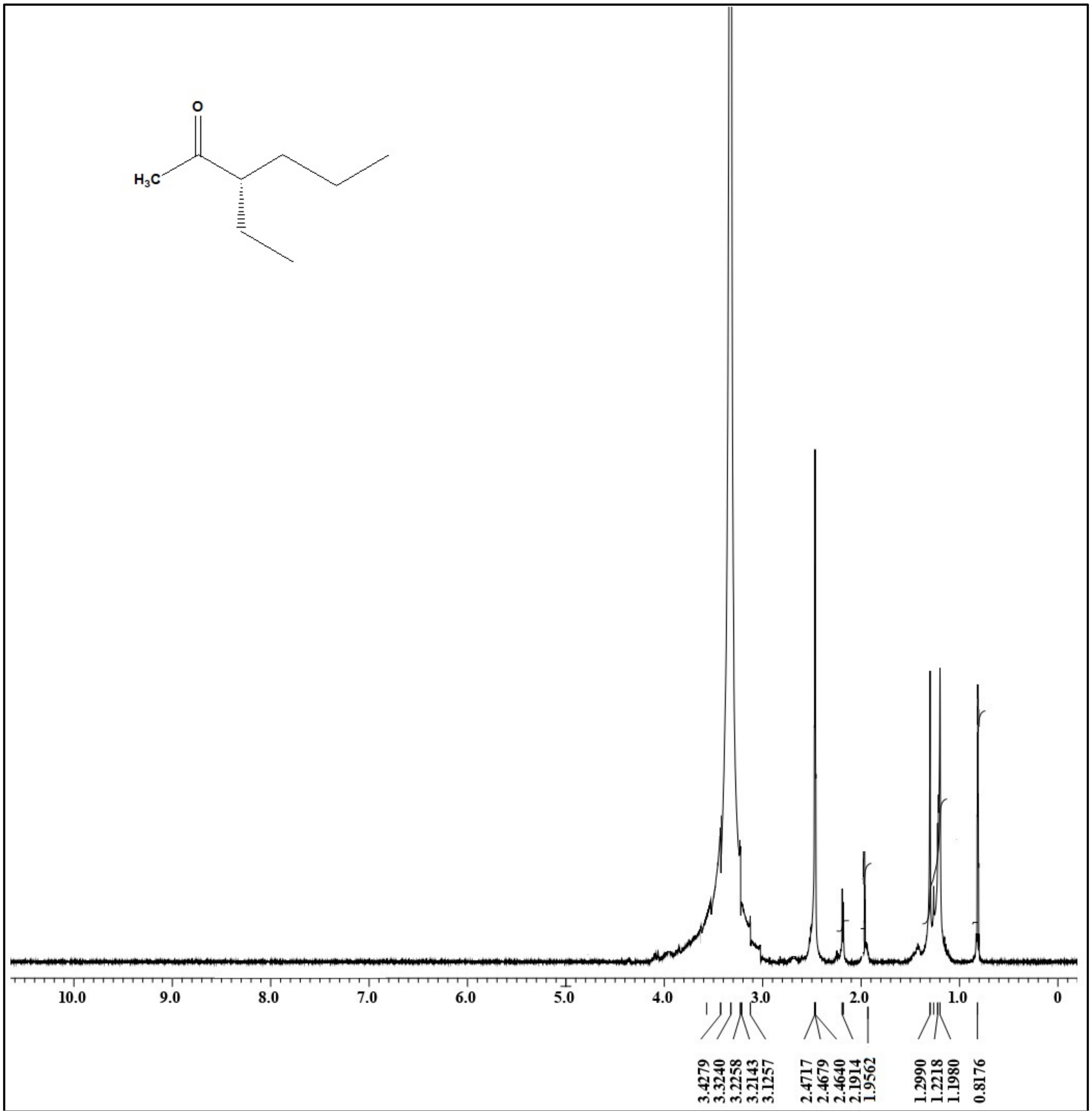

Figure S7: ^{1}HNMR spectra of compound 6a

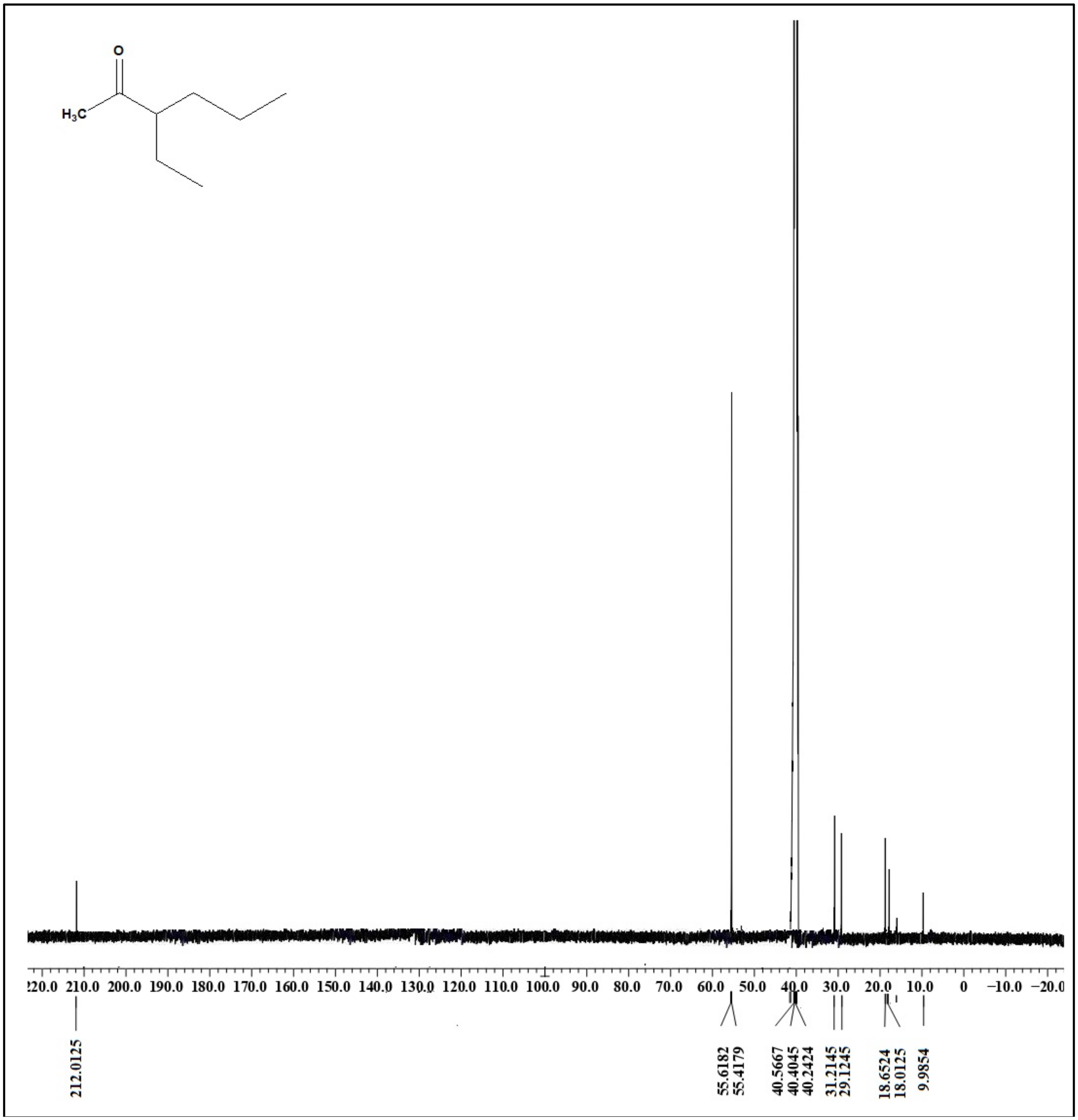

Figure S8: ^{13}CNMR spectra of compound 6a

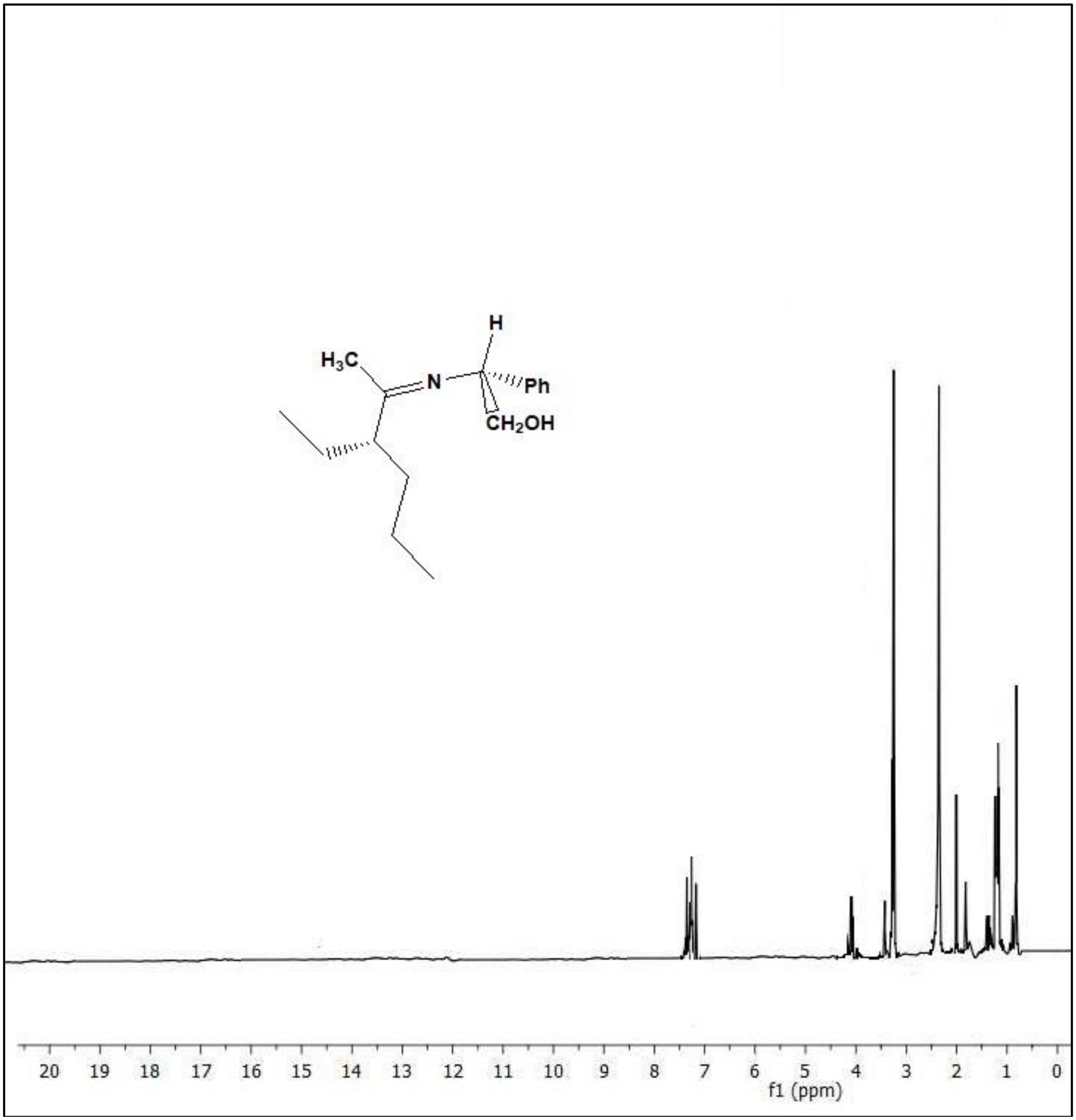

Figure S9: ^{1}HNMR spectra of compound 7a

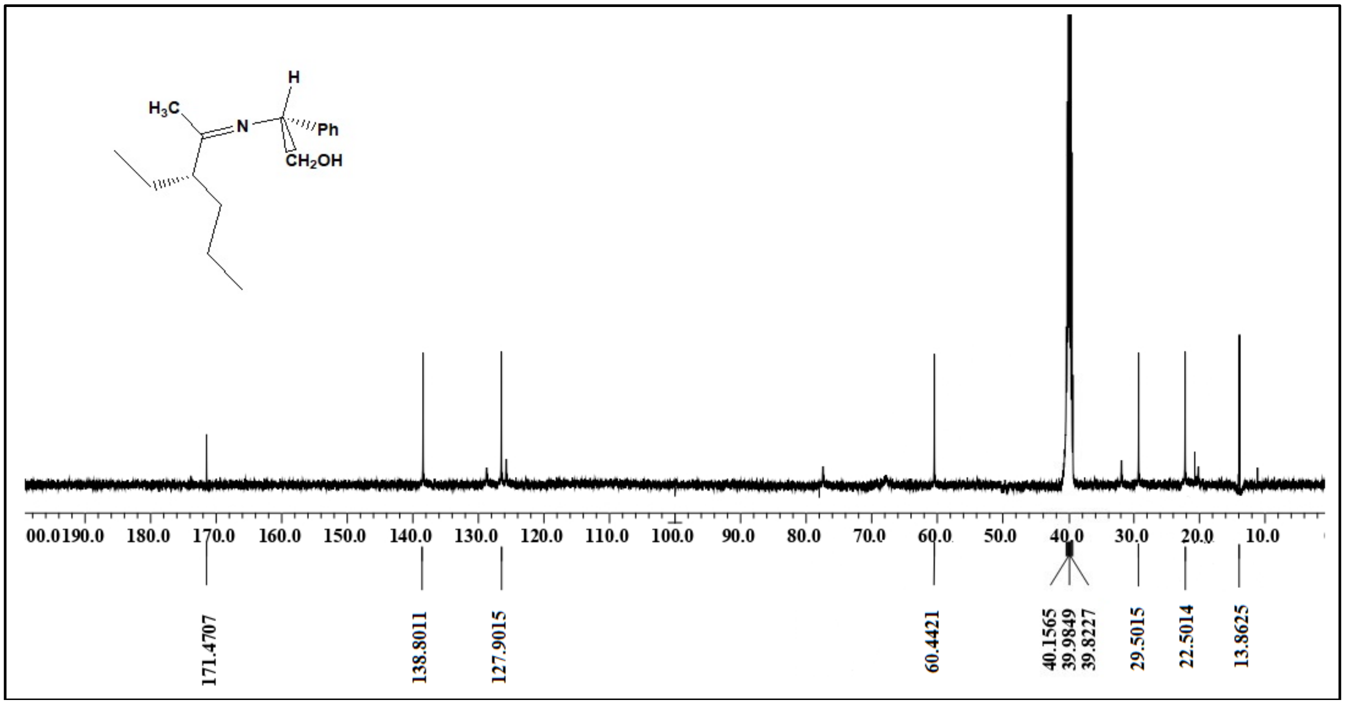

Figure S10: ^{13}CNMR spectra of compound 7a

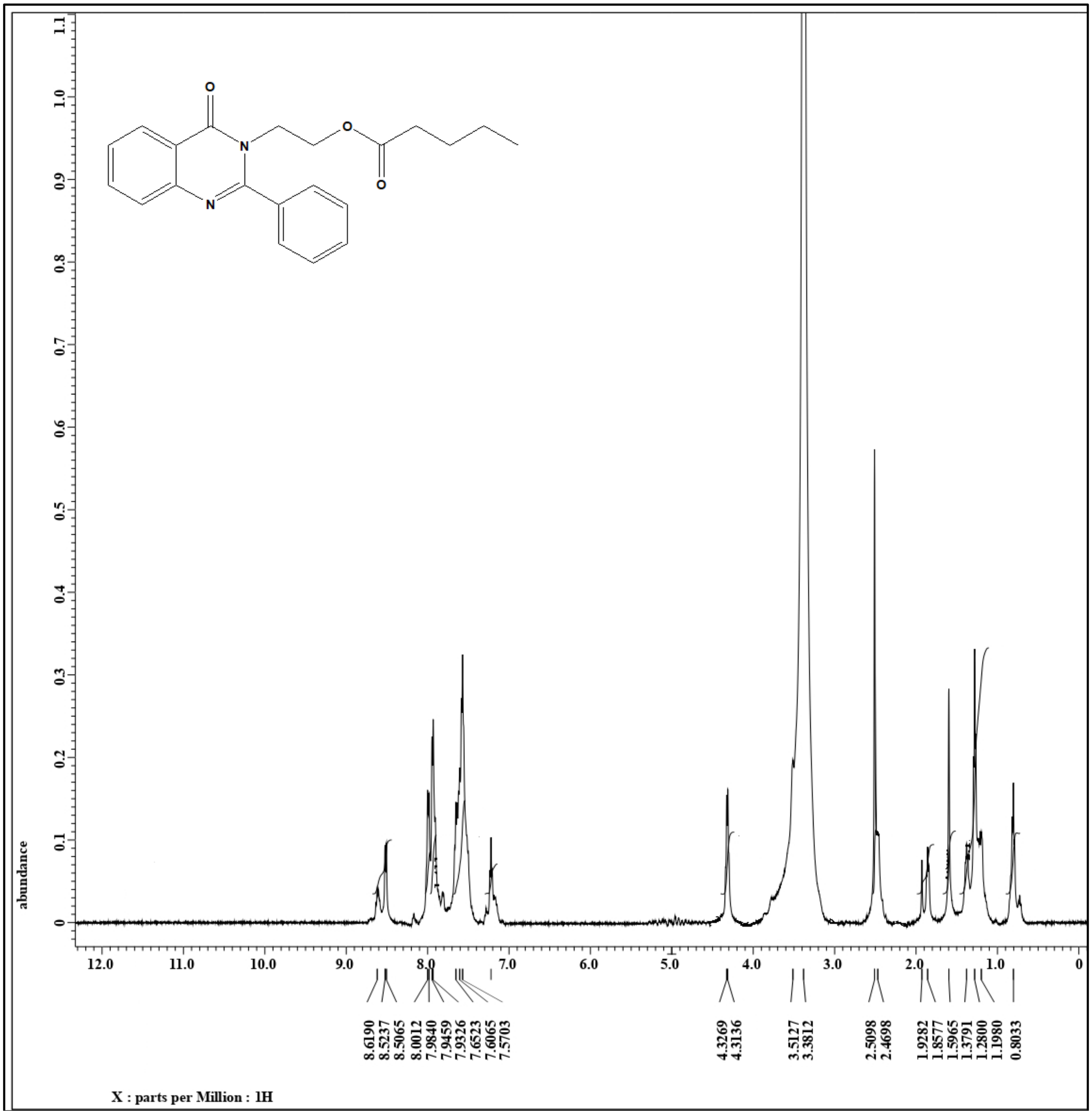

Figure S11: ^{1}HNMR spectra of compound 8a

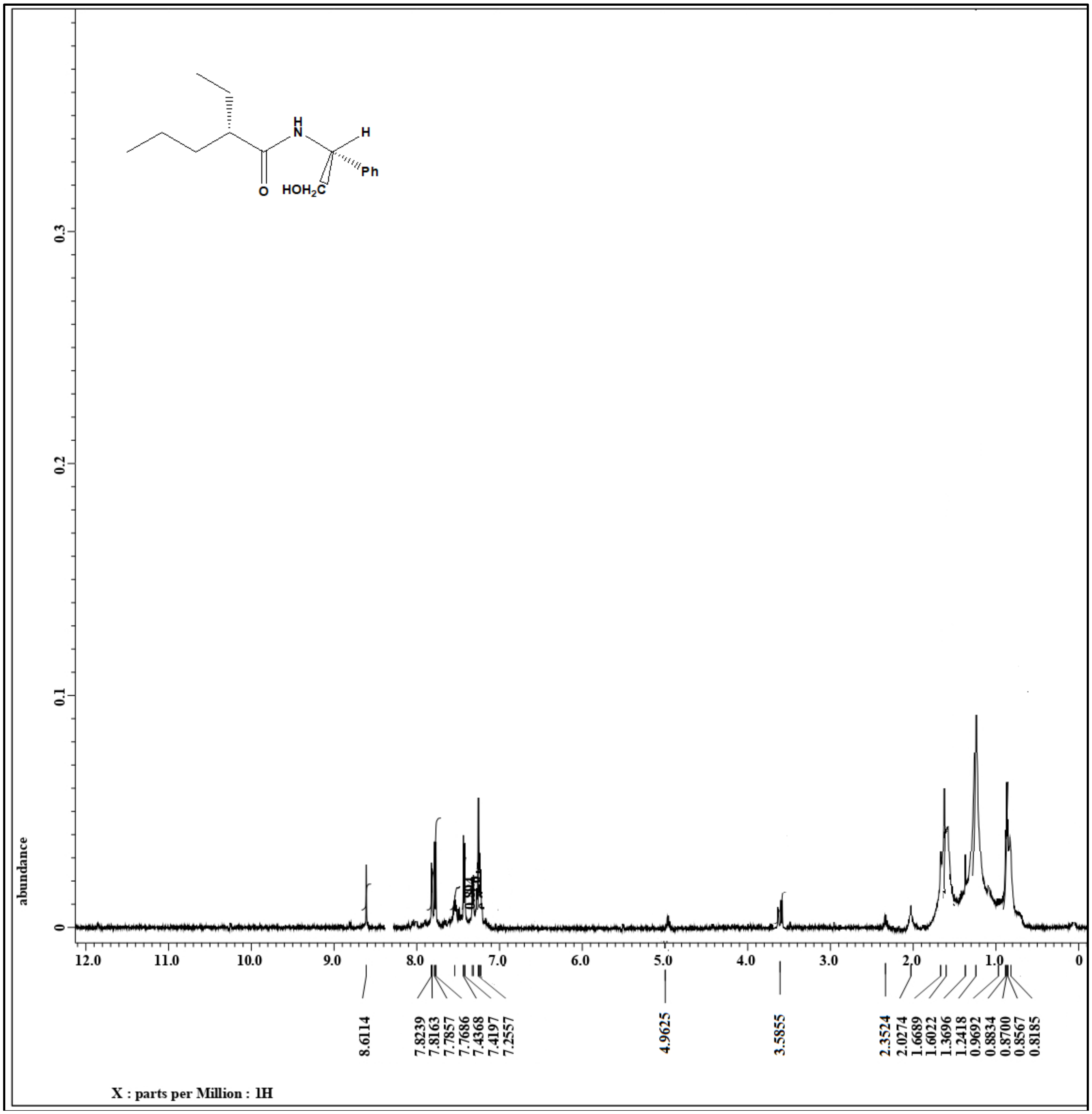

Figure S12: ^{1}HNMR spectra of compound 11a

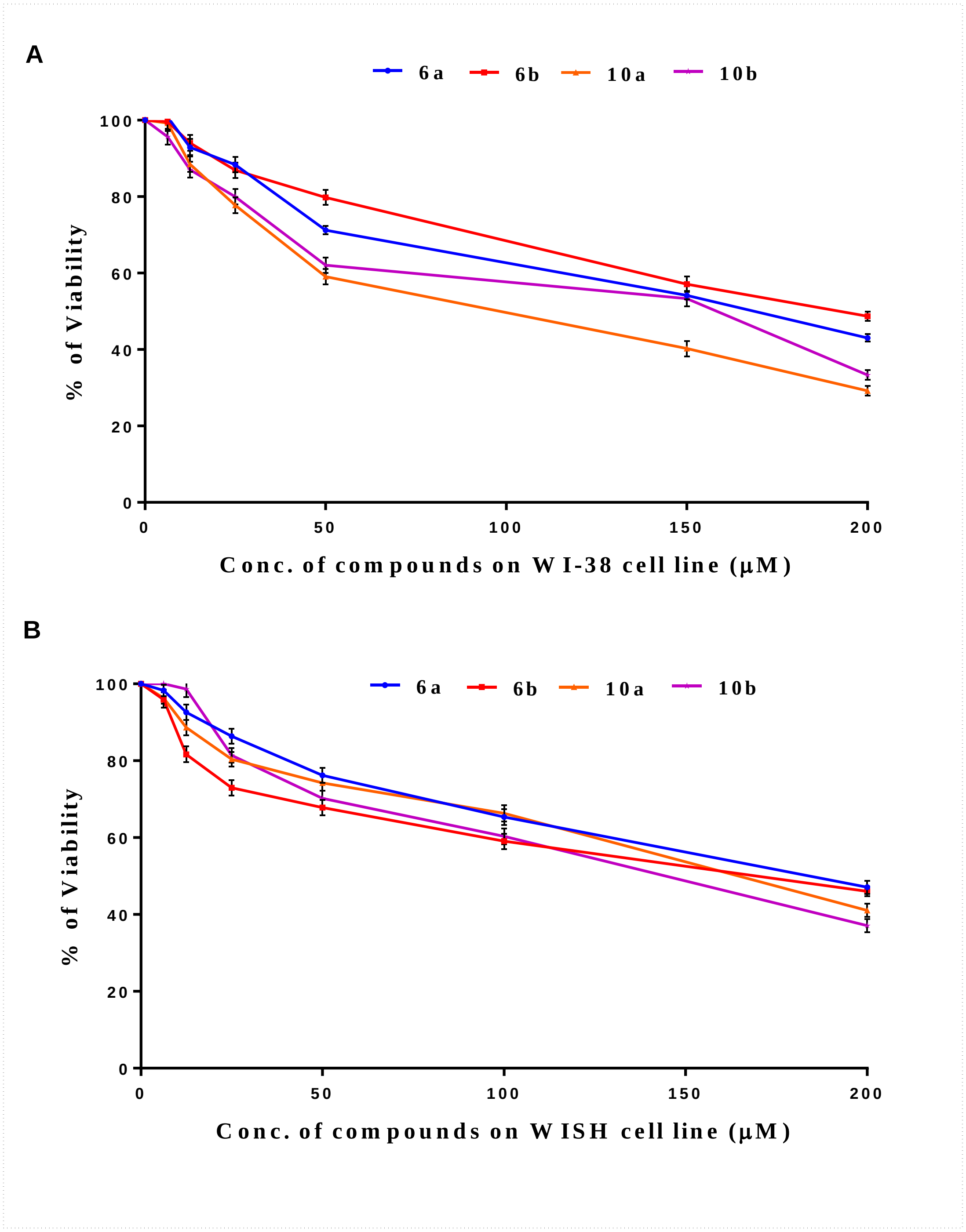

Figure S13: In-vitro MTT assay of all compounds on normal cell lines. [A] WI-38 cells, [B] WISH cells after 48h of incubation.

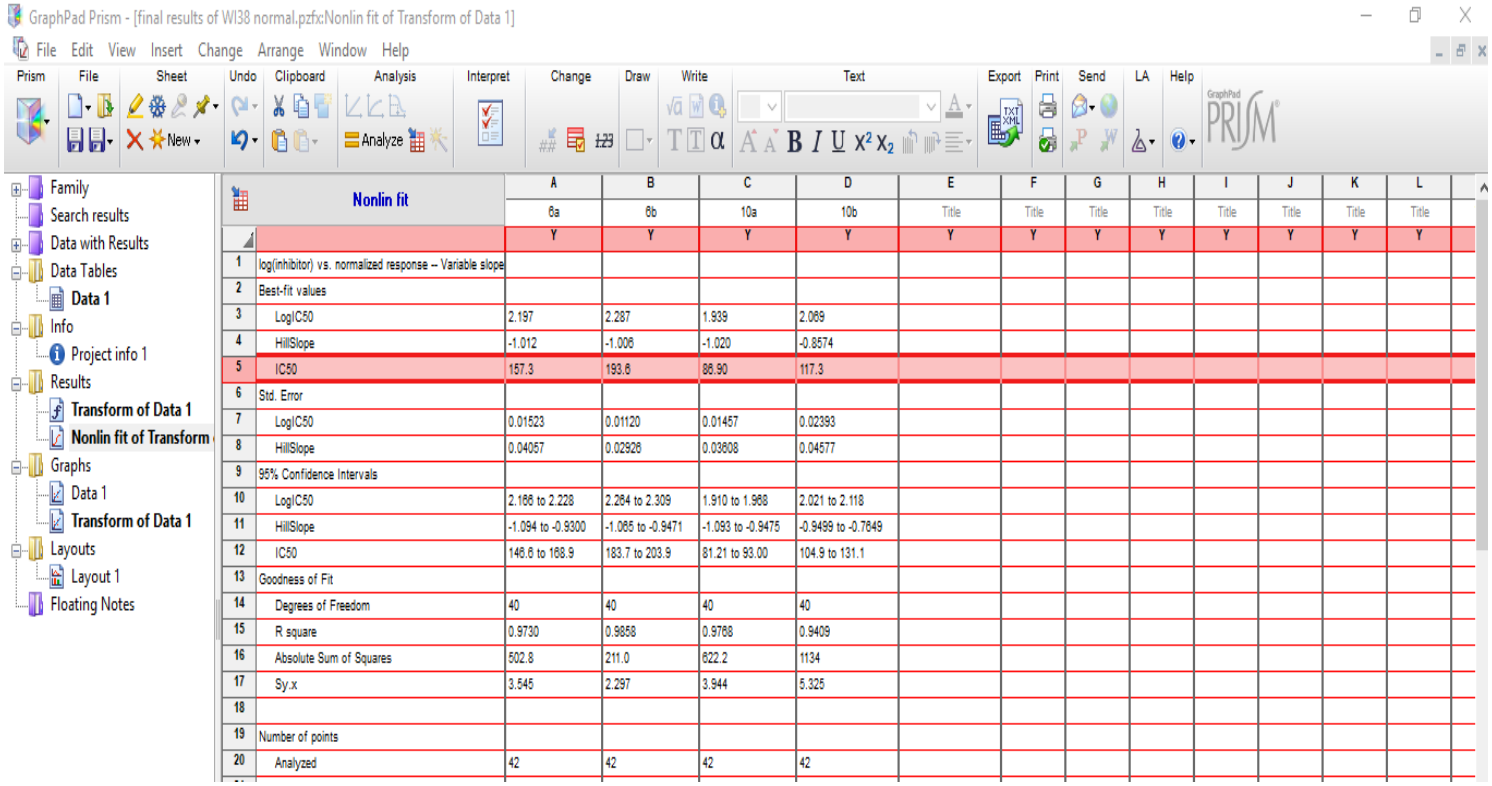

	Nonlin fit	A	B	C	D	E	F	G	H	I	J	K	L
		6a	6b	10a	10b	Title	Title	Title	Title	Title	Title	Title	Title
		Y	Y	Y	Y	Y	Y	Y	Y	Y	Y	Y	Y
1	log(inhibitor) vs. normalized response -- Variable slope												
2	Best-fit values												
3	LogIC50	2.197	2.287	1.939	2.069								
4	HillSlope	-1.012	-1.006	-1.020	-0.8574								
5	IC50	157.3	193.6	86.90	117.3								
6	Std. Error												
7	LogIC50	0.01523	0.01120	0.01457	0.02393								
8	HillSlope	0.04057	0.02926	0.03608	0.04577								
9	95% Confidence Intervals												
10	LogIC50	2.166 to 2.228	2.264 to 2.309	1.910 to 1.968	2.021 to 2.118								
11	HillSlope	-1.094 to -0.9300	-1.065 to -0.9471	-1.093 to -0.9475	-0.9499 to -0.7649								
12	IC50	146.6 to 168.9	183.7 to 203.9	81.21 to 93.00	104.9 to 131.1								
13	Goodness of Fit												
14	Degrees of Freedom	40	40	40	40								
15	R square	0.9730	0.9858	0.9768	0.9409								
16	Absolute Sum of Squares	502.8	211.0	622.2	1134								
17	Sy.x	3.545	2.297	3.944	5.325								
18													
19	Number of points												
20	Analyzed	42	42	42	42								

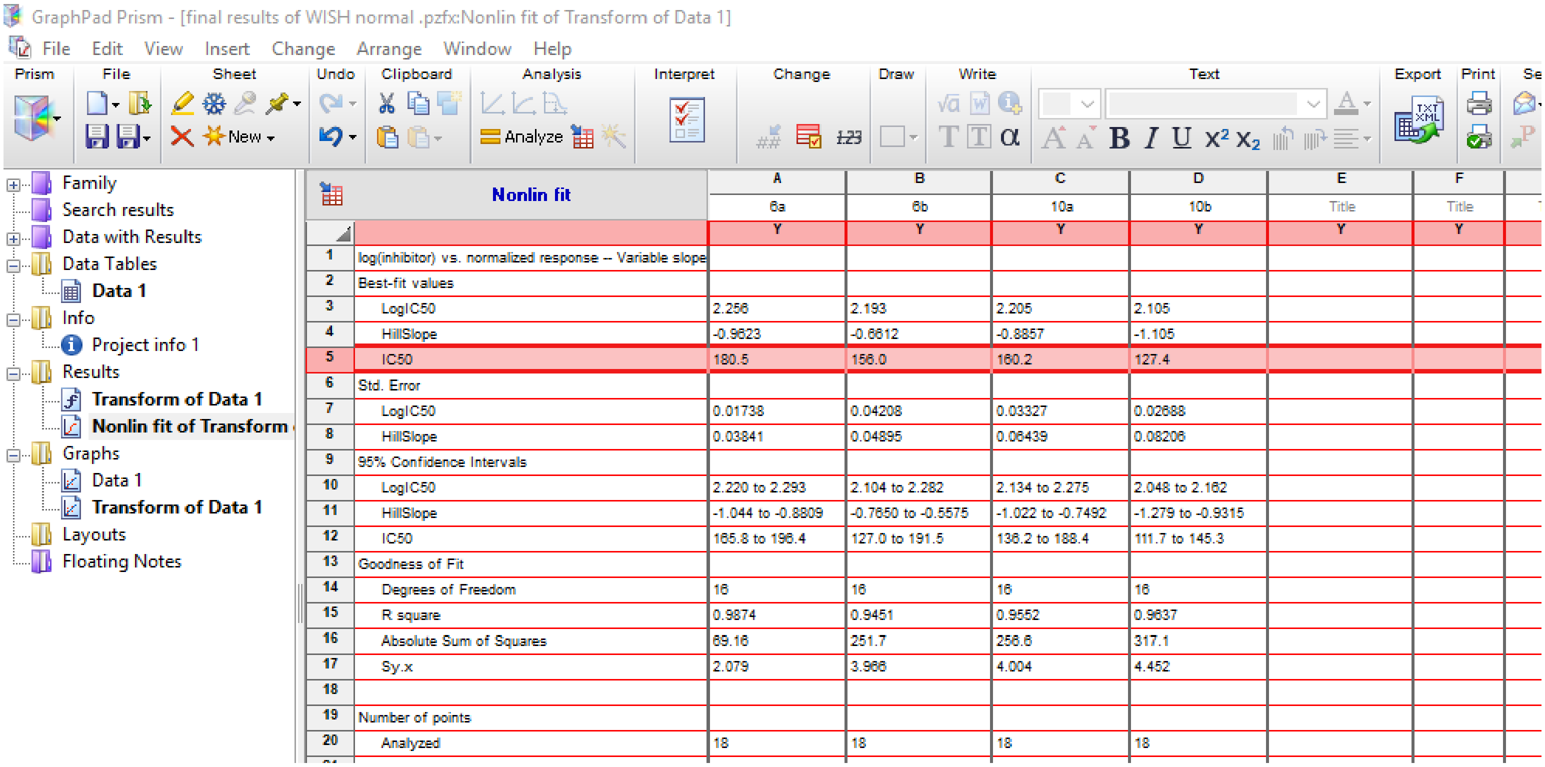

	Nonlin fit	A	B	C	D	E	F
		6a	6b	10a	10b	Title	Title
		Y	Y	Y	Y	Y	Y
1	log(inhibitor) vs. normalized response -- Variable slope						
2	Best-fit values						
3	LogIC50	2.256	2.193	2.205	2.105		
4	HillSlope	-0.9623	-0.6612	-0.8857	-1.105		
5	IC50	180.5	156.0	160.2	127.4		
6	Std. Error						
7	LogIC50	0.01738	0.04208	0.03327	0.02688		
8	HillSlope	0.03841	0.04895	0.06439	0.08206		
9	95% Confidence Intervals						
10	LogIC50	2.220 to 2.293	2.104 to 2.282	2.134 to 2.275	2.048 to 2.162		
11	HillSlope	-1.044 to -0.8809	-0.7650 to -0.5575	-1.022 to -0.7492	-1.279 to -0.9315		
12	IC50	165.8 to 196.4	127.0 to 191.5	136.2 to 188.4	111.7 to 145.3		
13	Goodness of Fit						
14	Degrees of Freedom	16	16	16	16		
15	R square	0.9874	0.9451	0.9552	0.9637		
16	Absolute Sum of Squares	69.16	251.7	256.6	317.1		
17	Sy.x	2.079	3.966	4.004	4.452		
18							
19	Number of points						
20	Analyzed	18	18	18	18		

Figure S14: The IC50 of all compounds on WI-38 and WISH normal cell lines
